# Investigation Into the Association Between Neurotransmitters, Immune Features, and Lung Adenocarcinoma: Identifying GABA‐Related Features Using Machine Learning Methods

**DOI:** 10.1155/sci/3060138

**Published:** 2026-05-21

**Authors:** Jiangtao You, Tianren Wang, Qingshi Wang, Yong Zhang, Rui Zhao, Wei Cui, Huan Chen

**Affiliations:** ^1^ Department of Thoracic Surgery, The First Affiliated Hospital of Xi’an Jiaotong University, Xi’an, Shaanxi, China, xjtu.edu.cn; ^2^ Department of Surgical Oncology, The First People’s Hospital of Xianyang, Xianyang, Shaanxi, China; ^3^ Department of Geriatric Endocrinology, The First Affiliated Hospital of Xi’an Jiaotong University, Xi’an, Shaanxi, China, xjtu.edu.cn

**Keywords:** gamma-aminobutyric acid, immunotherapy, lung adenocarcinoma, machine learning, neurotransmitters

## Abstract

**Background:**

Lung adenocarcinoma (LUAD), a predominant subtype of non‐small cell lung cancer (NSCLC), is associated with a high mortality rate. Currently, there are no reliable or sensitive biomarkers or prognostic methodologies available for its early detection or diagnosis. Gamma‐aminobutyric acid (GABA), a pivotal inhibitory neurotransmitter within the central nervous system (CNS), primarily exerts its effects through interactions with GABA receptors (GABARs). Recent studies have increasingly highlighted GABA’s significant role in mediating the initiation and progression of various tumors beyond the CNS. Nonetheless, research investigating the role of GABA in LUAD is limited, and the specific molecular and cellular mechanisms underlying its interactions remain to be fully elucidated.

**Methods:**

We developed an innovative machine learning framework designed to screen GABA‐related genes (GABARgenes) at both single‐cell and large transcriptomic levels. This framework encompasses 10 algorithms and 101 combinatorial pairing patterns, which facilitate the construction of consistent GABA‐related features (GABARFs). The framework’s performance was assessed using both a training set and an external validation set. To provide a quantitative prognostic tool for clinical application, we established a nomogram that incorporates GABARF. Additionally, we conducted multiomics analyses, including genomics, single‐cell transcriptomics, and comprehensive transcriptomics, to derive and consolidate more extensive prognostic features. We also evaluated the response of GABARF‐defined risk subgroups to immunotherapy and identified potential personalized therapeutic agents for specific risk categories.

**Results:**

Among the 124 GABARgenes analyzed, 38 demonstrated a significant correlation with overall survival (OS) in patients. Our machine learning–derived GABARF exhibited exceptional performance in predicting prognosis and clinical outcomes, showing promise in forecasting the onset and progression of LUAD. Multivariate analysis confirmed that GABARF serves as an independent prognostic factor for OS in LUAD. Furthermore, distinct GABARF risk subgroups exhibited significant differences in biological function, mutation status, and tumor immune infiltration. Notably, there were significant variations in the immunophenoscore (IPS) across the risk subgroups. GABARF risk stratification aligns with stemness properties of tumor cells, indicating that high‐risk patients may harbor tumors with enhanced stemness features that contribute to their poor prognosis and reduced immunotherapy response. Sensitivity analyses of conventional LUAD therapies indicated that patients in the low‐risk group may derive greater benefit from immune checkpoint inhibitors (ICIs), while those in the high‐risk group may exhibit heightened sensitivity to first‐line chemotherapy agents. Furthermore, LDHA overexpression was found to promote proliferation and migration, while inhibiting apoptosis. In addition, overexpression of LDHA can upregulate the expression of stemness markers CD133, SOX2, and OCT4 in LUAD cells, enhancing the malignant phenotype of tumor cells.

**Conclusion:**

This study presents a novel machine learning‐based model for GABARF, which shows promise as a potential tool to aid in prognostic prediction, targeted prevention, and individualized treatment planning in LUAD. Initial investigations into the interaction mechanisms of GABARF at the molecular, cellular, and tumor immune microenvironment (TIME) levels in LUAD have commenced. The GABARF model not only serves as a prognostic indicator but may also reflect the stemness status of LUAD tumors, offering insights into personalized treatment strategies that account for both neural‐immune‐stemness interactions.

## 1. Introduction

Lung cancer ranks as the leading cause of incidence and mortality among all malignant tumors worldwide, with lung adenocarcinoma (LUAD) representing the predominant pathological subtype, accounting for approximately 80%–85% of all lung cancer cases. This imposes a significant burden on the public health sectors globally [1]. The current treatment protocols for LUAD encompass traditional methods such as surgery, chemotherapy, and radiotherapy, alongside emerging targeted and immunotherapies. However, most of these treatment protocols fail to yield a favorable prognosis for patients with LUAD [2]. Traditional treatment protocols often fall short of achieving the desired outcomes. While targeted therapy proves beneficial for patients with specific driver gene mutations, challenges like drug resistance and persistent disease progression are inevitable with the prolonged usage of targeted drugs [3].

Indeed, the 5‐year survival rate for patients at TNM stage IA hits 60%, underscoring the significance of early diagnosis and intervention for an optimal prognosis in LUAD patients [4, 5]. Nonetheless, conventional diagnostic tools like CT/MRI, cytological examination, and tumor markers are suboptimal, leading to 75% of patients being in the late stage at diagnosis [5]. Consequently, the development of a sensitive and stable early diagnostic marker for LUAD becomes imperative. Moreover, the grim prognosis of LUAD is typically manifested in tumor recurrence and metastasis [2]. Therefore, the precision and reliability of early prognostic markers are essential for formulating personalized treatment strategies, reducing the likelihood of recurrence and metastasis, and extending patients’ disease‐free survival periods. The inherent heterogeneity of tumors presents a significant challenge in ensuring treatment efficacy. While immune checkpoint inhibitors (ICIs) can potentially reverse T cell exhaustion and effectively eliminate tumor cells, they often only benefit a minority of patients significantly and sustainably. Thus, our focus is directed toward discovering and identifying biomarkers or characteristics that are relevant to the prognosis of LUAD patients and their responses to ICI treatment.

Gamma‐aminobutyric acid (GABA) acts as an essential inhibitory neurotransmitter in the central nervous system (CNS) of various mammals, including humans. In the CNS, glutamate, which functions as a precursor of GABA, contributes to GABA’s production and release, aided by the GAD enzyme [6]. Most tumors exhibit a significant dependence on glutamate due to its vital role in tumor energy metabolism [7]. Simultaneously, GABA concentrations are elevated in several solid tumors outside the CNS, like lung [8], colon [9], and stomach [10] cancer, and are associated with cancer progression [11]. GABA performs cellular regulation by interacting with specific receptors. It encourages the growth and migration of tumor cells when bound to GABAA receptors (GABAARs), but it can suppress tumor development when it interacts with GABAB receptors [12]. Moreover, recent studies have hinted that neurotransmitter signaling, including GABA, may critically regulate the stemness properties of tumor cells, driving drug resistance and tumor relapse. These processes are associated with traditional signaling pathways like MAPK/ERK [12]. Furthermore, it has been reported that B cells can produce GABA to facilitate the generation of M2 macrophages, consequently diminishing the responsiveness of tumor cells to CD8^+^ T cells [13]. There appears to be a consensus across multiple disciplines that GABA, whether directly or indirectly through immune mechanisms, plays a role in both the initiation and progression of tumors. However, there is limited research investigating GABA’s potential as a quantitative marker for the clinical prognosis of LUAD or its capacity to assess patient susceptibility and response to immunotherapy or chemotherapy.

This study employs multiomics analysis techniques to investigate the immunological and molecular characteristics of GABA in LUAD. GABA‐related genes (GABARgenes) were successfully identified using single‐cell and transcriptome sequencing technologies. An accurate predictive model, referred to as GABA‐related features (GABARFs), was constructed using an innovative bioinformatics approach that integrates 10 machine learning algorithms and their 101 combinations.

The accuracy of GABARF in predicting the occurrence, progression, and metastasis of LUAD was assessed, and a nomogram was developed for quantitative prognosis analysis of patients. From both genomic and transcriptomic perspectives, the molecular and cellular mechanisms associated with GABARF were explored to elucidate the relationship between GABARF and LUAD prognosis, as well as the status of the tumor immune microenvironment (TIME). Additionally, the sensitivity of different GABARF risk subgroups to ICIs and first‐line chemotherapy drugs was analyzed.

The primary aim of this study is to clarify the predictive capacity of GABA for the prognosis of LUAD patients and to explore its potential role in guiding the development of personalized immunotherapy strategies.

## 2. Methods

### 2.1. Cell Culture and Transfection

The human LUAD cell line A549 was obtained from the American Type Culture Collection (ATCC). Cells were cultured in Roswell Park Memorial Institute (RPMI)−1640 medium supplemented with 10% fetal bovine serum (FBS) and 1% penicillin–streptomycin. All cell cultures were maintained in a humidified incubator at 37°C with a 5% CO_2_ atmosphere. For transfection, A549 cells were seeded in 6‐well plates and grown to 70%–80% confluence, after which they were transfected with LDHA overexpression plasmids or empty vector controls using Lipofectamine 3000 reagent (Invitrogen) according to the manufacturer’s protocol.

### 2.2. Source and Processing of Experimental Data

We compiled RNA‐seq data from TCGA for LUAD patients, including gene expression profiles and clinical data. We analyzed transcripts with TPM values above 0.1 and excluded genes with lower average expression. We also retrieved the GSE149655 single‐cell RNA‐seq dataset from GEO, containing normal and LUAD tumorous tissue samples. Copy number variation (CNV) data for TCGA‐LUAD patients was obtained from UCSC Xena. To assess the impact of GABA scores on disease prediction, we used datasets GSE31210 and GSE500811 from GEO. The R package “imvigor210corebiologs” was utilized to analyze the IMvigor210 series, aiming to validate prognostic markers’ effectiveness in predicting patient response and changes post‐immunotherapy.

Single‐cell RNA‐seq data was analyzed using the R package, with the raw data from nine LUAD patients in the GSE149655 dataset being processed and analyzed. Initially, cell subgroups were constructed using the “FindNeighbors” and “FindClusters” functions and were visualized using the “t‐SNE” method. The “Harmony” package was also utilized to mitigate batch effects in the sample data. Subsequently, dimensionality reduction and annotation were performed on cells based on marker genes of different cell types. Concurrently, in order to identify GABARgenes, 128 such genes from previous reports were collated [14]. Based on this, a high‐expression and low‐expression gene spectrum of GABA in various related cells was constructed using the “limma” function of the R package. The raw expression matrix was processed with R software, and the analysis was continued using the “limma” package to screen for differentially expressed genes (DEGs) between the two groups. For DEG identification, we applied filtering criteria of *p*  < 0.05 and |LogFC| > 0.5. The *p*‐value threshold of 0.05 with Wilcoxon test was used to control the false discovery rate, as this threshold balances statistical stringency with biological relevance in single‐cell transcriptomic studies. The fold‐change threshold of |LogFC| > 0.5 was selected to identify genes with meaningful biological differences while maintaining adequate sensitivity for detecting moderate expression changes that may be functionally relevant in GABA‐related pathways. Genes with differential expression levels were considered to be involved in GABA expression or molecular biological activities at the single‐cell transcriptome level, and these genes were included in the subsequent weighted gene co‐expression analysis (WGCNA). The R package “CellChat” was also utilized for cell interaction analysis.

During quality control of single‐cell RNA‐seq data, we applied a mitochondrial gene percentage threshold of <20% to filter out low‐quality or apoptotic cells. While a 5% threshold is commonly used as a default in many scRNA‐seq software packages, this value was originally established for healthy tissues and may be overly stringent for certain tissue types. The mitochondrial content varies substantially across tissues based on their metabolic demands, with energy‐intensive tissues naturally exhibiting higher mitochondrial transcript proportions. Lung tissue, which has moderate‐to‐high energy requirements, often displays elevated baseline mitochondrial RNA content. Recent systematic analyses of over 5.5 million cells across 1349 datasets have demonstrated that mitochondrial percentage thresholds should be tissue‐specific and data‐adaptive rather than universally fixed at 5%. A threshold of 20% for LUAD samples allows retention of viable cells with naturally higher mitochondrial content while effectively removing dying or stressed cells, thereby preventing the inappropriate exclusion of biologically relevant cell populations

### 2.3. Single‐Sample Gene Set Enrichment Analysis (ssGSEA) and Gene Set Enrichment Analysis (GSEA)

ssGSEA is commonly used to measure the relative abundance of cell types in a sample by comparing gene expression data against a cell gene set. In our study, we applied ssGSEA from the “GSVA” package in R to analyze tumor and control samples, calculating the GABA score for each and assessing the enrichment of various cell types. This technique helps evaluate relative cell expression. We also performed GSEA to identify biological processes (BPs), cell composition, and molecular functions (MFs) in different risk subgroups, including the significantly enriched HALLMARK gene set in the high‐risk subgroup. To explore pathways related to signal transmission, we calculated GSVA scores for 50 hallmark pathways and used the “limma” package to analyze differences between high‐risk and low‐risk groups. By examining pathway correlations, we selected top pathways to investigate their association with disease prognosis.

### 2.4. WGCNA of GABA‐Associated Genes in LUAD

This study uses the WGCNA package in R to build a gene co‐expression network. WGCNA, a systems biology approach, is applied to map gene associations across various samples, identifying groups of genes with high covariance. Hierarchical clustering was performed to eliminate outliers, and gene pairing relationships were determined based on GABARgene expression levels. The Pearson correlation coefficient was calculated for gene pairs, and a similarity matrix was created. The adjacency matrix was then converted into a topological overlap matrix (TOM) to detect gene connectivity in the network, and dissimilarity (dissTOM) was computed. Genes were categorized into clusters or modules based on connectivity and covariance, followed by hierarchical clustering of these clusters. Dynamic tree cutting was used to form co‐expression modules, grouping highly correlated genes together. The module most correlated with GABA scoring was identified for further analysis.

In the WGCNA analysis, we set the minimum module size to 60 genes. This parameter determines the smallest number of genes that can form a distinct co‐expression module. The choice of minimum module size represents a balance between statistical robustness and biological interpretability. Smaller modules (e.g., <30 genes) may represent spurious correlations or lack sufficient statistical power, while overly large minimum sizes may merge distinct biological pathways into single modules, reducing biological resolution. A minimum module size of 30–100 genes is recommended in the WGCNA literature and widely adopted across diverse applications, including cancer genomics, neurodegeneration, and cardiovascular disease studies. We selected 60 genes, as this threshold provides sufficient statistical power to detect reliable co‐expression patterns while maintaining the ability to identify functionally distinct biological modules relevant to GABA signaling pathways in LUAD.

### 2.5. The Prediction Model Was Constructed by Integrating Machine Learning

We employed WGCNA to pinpoint modules highly correlated with GABA at the RNA‐seq level, and utilized the R package “limma” to analyze the DEGs between tumor and normal tissues. Subsequently, we undertook a cross‐analysis of the DEGs with the genes present in the GABA‐related modules, as identified by WGCNA, and the intersecting genes were deemed to contribute to GABA synthesis at the single‐cell transcriptome level. As a result, we labeled these genes as GABARgenes. To construct a reliable and effective prognostic model, we implemented the following steps: initially, we employed univariate Cox regression analysis to screen for GABARgenes with prospective prognostic value in the dataset. The TCGA‐LUAD dataset was set as the training set, and the GSE31210 and GSE500811 datasets were set as external validation sets. To construct a variable selection and model based on a 10‐fold cross‐validation framework, we employed 101 combinations of these 10 algorithms in the training dataset (TCGA‐LUAD dataset). This process utilizes the R package Mime to accomplish. Prior to generating the 101 combinatorial frameworks, the prognostic capability of each of the 10 individual machine learning algorithms was independently evaluated. Given the time‐to‐event nature of our overall survival (OS) data with right‐censoring, Harrell’s concordance index (C‐index) was employed as the primary specific performance metric to systematically assess the individual baseline algorithms, as traditional binary classification metrics (e.g., precision, recall, and F1‐score) are not statistically appropriate for handling censored survival data. Following this, we assessed all models in the external validation sets (GSE31210 and GSE500811 datasets), and selected the algorithm combinations with sturdy performance and significant clinical translatability. Ultimately, we established a final feature termed GABARF to predict the OS of lung cancer patients.

### 2.6. Survival Analysis and Nomogram Construction

Utilizing the median GABARF risk score, the TCGA‐LUAD (training set) and the external validation set were stratified into high‐risk and low‐risk categories based on this score. The “survminer” package within R was utilized to execute KM survival curve analysis to determine whether a significant disparity in OS existed between the high‐risk and low‐risk groups (log‐rank test, *p*  < 0.05). Concurrently, an analysis was conducted to understand the correlation between GABARF and certain clinical features (such as age, sex, TMN staging, and grading). Subsequently, a nomogram was developed that integrates GABARF and clinical features to quantify the anticipated survival time of LUAD patients, with the aim of enhancing the accuracy of the model’s prognosis. The clinical decision curve analysis (DCA) was utilized to evaluate the net clinical benefit of the model, and the precision and accuracy of the nomogram were assessed through the receiver operating characteristic (ROC) and calibration curve.

### 2.7. Analysis of Genomic Variation Among GABA Risk Subgroups

Mutant‐allele tumor heterogeneity (MATH) is a method to measure gene diversity in tumors using mutant allele frequency. It has been acknowledged for its predictive value in different cancer types. MATH values are derived from sequencing the tumor and normal tissue exomes, indicating tumor heterogeneity levels. This study analyzed lung cancer survival based on MATH values and used “maftools” R package to show mutation status in LUAD patients’ risk subgroups through waterfall plots. Additionally, 30 genes with significant differences between high and low‐risk subgroups were chosen for CNV analysis.

### 2.8. Comprehensive Analysis of Immune Profile and Response to ICI Therapy

We utilized the CIBERSORT algorithm to quantify the infiltration of 14 types of immune cells, aiming to explore the association between GABARF and immune infiltration. The anti‐tumor immune cycle serves as the fundamental logical framework informing the application of immunotherapy. It involves seven basic steps: the release of cancer cell antigen, antigen presentation, T cell activation, T cell migration, T cell infiltration into tumor tissue, T cell recognition, and elimination of cancer cells. We obtained the cancer‐killing immune step activity score from the tumor immune phenotype (TIP) tracking platform (http://biocc.hrbmu.edu.cn/TIP/index.jsp). To evaluate immunogenicity, which is based on immunomodulators, immunosuppressive cells, MHC molecules, and effector cells, we implemented algorithms utilizing machine learning techniques to calculate an immunophenoscore (IPS) score from unbiased gene expression of representative cell types. The IPS score exhibited a positive correlation with the efficacy of immunotherapy. We retrieved IPS scores of TCGA‐LUAD patient samples from The Cancer Immunome Atlas (TCIA) database.

### 2.9. Prediction of GABA Drug Sensitivity

Currently, numerous clinical healthcare organizations are actively advocating for individualized therapy. As a result, we employed the “pRRrophetic” package in R language to predict the drug susceptibility of lung cancer patients based on varying GABARF risk ratings. This method achieves the objective of calculating the half‐maximal inhibitory concentration (IC50) by aligning the gene expression spectrum of LUAD tissues with that of normal tissues. Using the Wilcoxon test, we examined the differences in drug IC50 between the high‐risk and low‐risk groups, considering a *p*‐value less than 0.05 as statistically significant.

### 2.10. Apoptosis Assay

Apoptosis was evaluated 72 h post‐transfection. Cells were detached using trypsin without EDTA and subsequently centrifuged at 300 g for 5 min at 4°C to facilitate collection. The resulting cell pellet was washed twice with PBS. After discarding the supernatant, the cells were resuspended in 100 μL of 1× binding buffer. For staining, 5 μL of Annexin V‐FITC and 10 μL of PI staining solution were added to the suspension, followed by gentle mixing. The cell suspension was incubated in the dark at room temperature for 10–15 min. Following incubation, 400 μL of diluted 1× binding buffer was added to the samples, which were then mixed thoroughly before flow cytometric analysis.

### 2.11. Wound‐Healing Assay

Cells were transfected with plasmids for 24 h and subsequently scratched using a pipette tip to create a wound. The monolayer was washed once with PBS and then replenished with complete culture medium. Initial images of the wound were captured immediately after washing, and the cells were incubated for an additional 72 h to facilitate migration and proliferation into the wounded area.

### 2.12. Cell Proliferation Assay

Cell viability and proliferation were evaluated utilizing the cell counting kit‐8 (CCK‐8) assay (Dojindo, Japan). Briefly, transfected A549 cells were harvested and seeded into 96‐well plates at a density of 2 × 10^3^ cells per well in 100 μL of complete culture medium. At designated time points (0, 24, 48, and 72 h post‐seeding), 10 μL of CCK‐8 reagent was added to each well, followed by an additional incubation in the dark at 37°C for 2 h. The optical density (OD) was subsequently measured at a wavelength of 450 nm using a microplate reader to quantify cellular proliferation.

### 2.13. Western Blot Analysis

Total proteins were extracted from A549 cells using RIPA lysis buffer supplemented with protease and phosphatase inhibitor cocktails, and protein concentrations were quantified using a BCA protein assay kit. Equivalent amounts of protein lysates (30 μg) were resolved by 10% sodium dodecyl sulfate–polyacrylamide gel electrophoresis (SDS–PAGE) and electro‐transferred onto polyvinylidene fluoride (PVDF) membranes. After blocking with 5% non‐fat milk in tris‐buffered saline containing 0.1% Tween‐20 (TBST) for 1.5 h at room temperature, the membranes were incubated overnight at 4°C with primary antibodies targeting LDHA, CD133, SOX2, OCT4, and GAPDH (internal control). Following incubation with horseradish peroxidase (HRP)‐conjugated secondary antibodies for 1 h at room temperature, the protein bands were visualized using an enhanced chemiluminescence (ECL) detection system and analyzed with ImageJ software.

### 2.14. Statistical Analysis

All experimental results were statistically analyzed using R software. We assessed the significance of DEGs using FDR‐corrected *p*‐values. To assess the model*’s* performance, we plotted the ROC curve and calculated the area under the curve (AUC). We conducted Kaplan–Meier survival analysis and log‐rank tests to compare the OS of patients in different subgroups. Additionally, we performed univariate and multivariate Cox regression analyses in parallel to identify independent prognostic factors. We considered a *p*‐value less than 0.05 to indicate statistical significance.

## 3. Results

### 3.1. Distribution Characteristics of GABA Activity in Single‐Cell Transcriptomes

**Figure 1:**
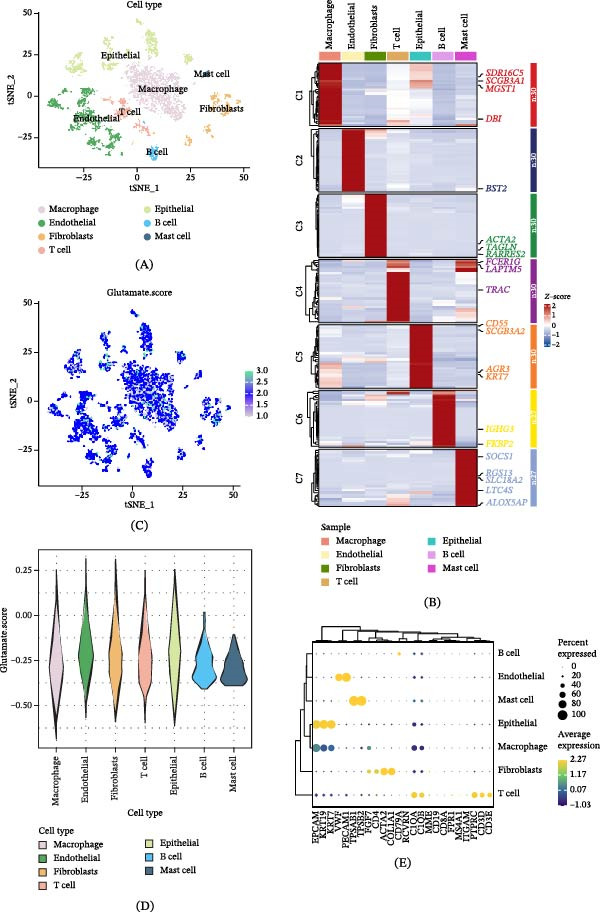
Characteristics of gamma‐aminobutyric acid (GABA) in the single‐cell transcriptome. (A) The t‐SNE plot delineates the cell types discerned by marker genes. (B) The heatmap manifests the prominent marker gene clusters in each cell. (C) Activity scores of gamma‐aminobutyric acid (GABA) for each cell. (D, E) Dispersion of GABA scores across diverse cell types.

Single‐cell RNA‐seq data from four LUAD patients was consolidated, followed by performing dimensionality reduction and clustering analysis of cells based on the initial 2000 variant genes (Figure 1A). Cells were initially categorized into seven subgroups using distinct cell marker genes, including macrophages, endothelial cells, fibroblasts, T cells, epithelial cells, B cells, and adipocytes, as illustrated in a heatmap depicting the marker genes of each cell subgroup (Figure 1B). The “AddModuleScore” feature in the Seurat package was utilized to quantify the expression levels of 124 GABARgene sets across all cell types, thereby depicting the activity of GABA in various cell types (Figure 1C). The findings suggested a notably higher GABA activity in endothelial cells, epithelial cells, and fibroblasts compared to other cells among these seven cell types (Figure 1D,E). The cells were partitioned into high‐activity and low‐activity groups based on the GABA activity, followed by an examination of the 518 DEGs between the two groups for further analysis.

### 3.2. Identification of Highly Correlated Modules and Screening of GABARgenes

We employed the WGCNA approach to construct co‐expression networks, fitting the model based on the scale‐free topology criterion. We set the scale‐free fitting index to 0.85, thereby determining the minimum soft‐thresholding value to be 7 (i.e., the optimal threshold) to maintain scale‐free topology and effective connectivity. We built the co‐expression network using 518 DEGs identified from single‐cell transcriptomics. We constructed the network according to the optimal threshold of 7, combining modules with an eigengene value greater than 0.75 (setting the minimum number of genes in the module to 60), yielding four co‐expression modules (Figure 2A–C). Among them, the turquoise module had the highest correlation with the GABA score (*r* = 0.4, *p* = 3e − 24), thus we selected it as the core gene module with high correlation. Furthermore, we assessed the relationship between module membership (MM) and gene significance (GS) in the core turquoise module, where MM denotes the correlation between module gene expression values and ME values, and GS represents the correlation between module genes and the sample. The MM vs. GS graph shows a correlation coefficient of 0.4 between the GABA score and the turquoise module, with a corresponding *p*‐value of 2e − 05 (Figure 2D), suggesting that the genes within this module may have significant GABA‐related function. The expression differences of DEGs between tumor and normal lung tissues are displayed in a volcano plot (Data source: TCGA‐LUAD) (Figure 2E). Of the 107 genes in the turquoise module, 100 are intersecting genes with DEGs (Figure 2F). We refer to these genes as GABARgenes, which may be involved in the molecular expression or response process of GABA or its receptors at the whole and single‐cell transcriptomic levels. We performed GO enrichment analysis on GABARgenes to study the BPs of potential gene targets. GO enrichment analysis revealed that these targets have various functions, such as protein macromolecule activity, and so on. Moreover, these genes are related to various cellular components (CCs), such as cell substrate junction, focal adhesion, et cetera. Our analysis also showed a significant correlation between the genes in the key module and MFs, including the regulation of peptidase activity (Figure 2G). After conducting a univariate Cox regression analysis on these 100 intersecting genes, we identified 38 significant genes with *p*‐values less than 0.05. Further, based on TCGA and external validation sets, we selected 38 shared genes related to prognosis. We presented the results of the univariate Cox regression analysis and the interrelation among these 38 genes (Figure 2H). Additionally, we analyzed the CNVs of these 38 genes, among which the copy number increase frequencies of YWHAZ and EIF4A3 both exceeded 10% (Figure 2I).

Figure 2Identification of the gamma‐aminobutyric acid related gene (GABARgene). (A) The dendrogram illustrates the hierarchical clustering of TCGA‐LUAD samples. (B) The lower heat map represents the GABA score of each sample, as calculated by the ssGSEA algorithm. (C) The dendrogram shows the cluster analysis conducted by WGCNA. (D) The heat map indicates a close relationship between the turquoise module and ICD traits. (E) The scatterplot demonstrates the relationship between gene significance (GS) and module membership (MM) within the turquoise module. (F) The volcano plot presents the results of differential analysis between normal and tumor samples of TCGA‐LUAD, with the top five upregulated and downregulated genes distinctly marked. (G) The Venn diagram displays the intersecting genes between the turquoise module and DEGs in extensive RNA‐seq data. (H) GO enrichment analysis of GABARgenes. (I) The results of univariate Cox regression analysis and gene correlations of GABARgenes.

**Figure:**
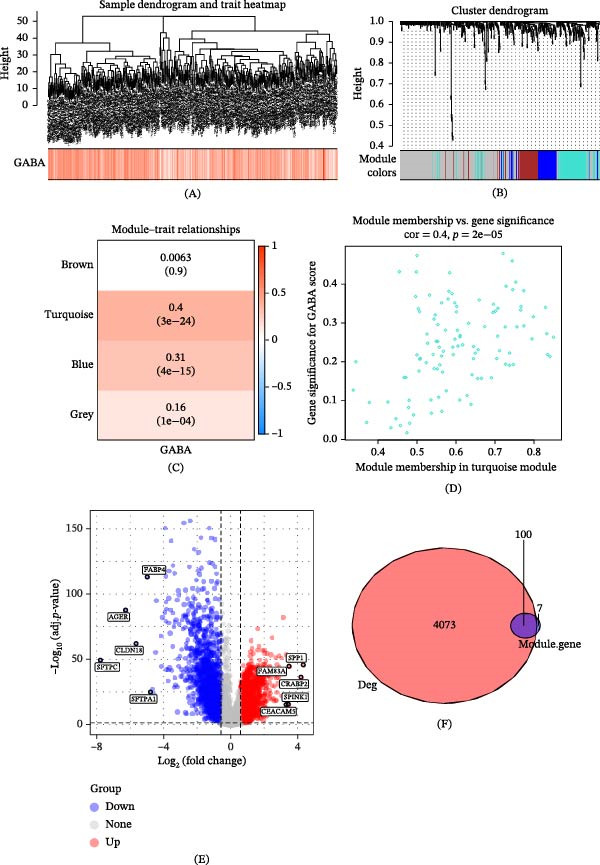

**Figure:**
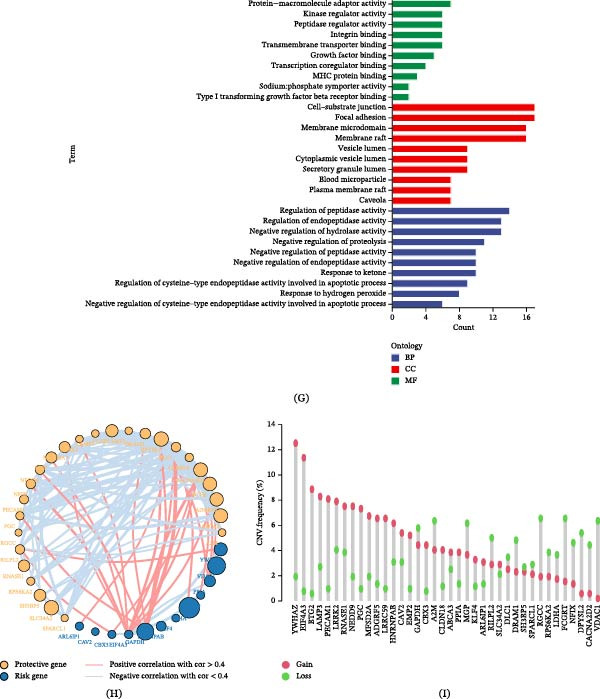

### 3.3. To Develop Prognostic Models Based on Machine Learning

**Figure 3:**
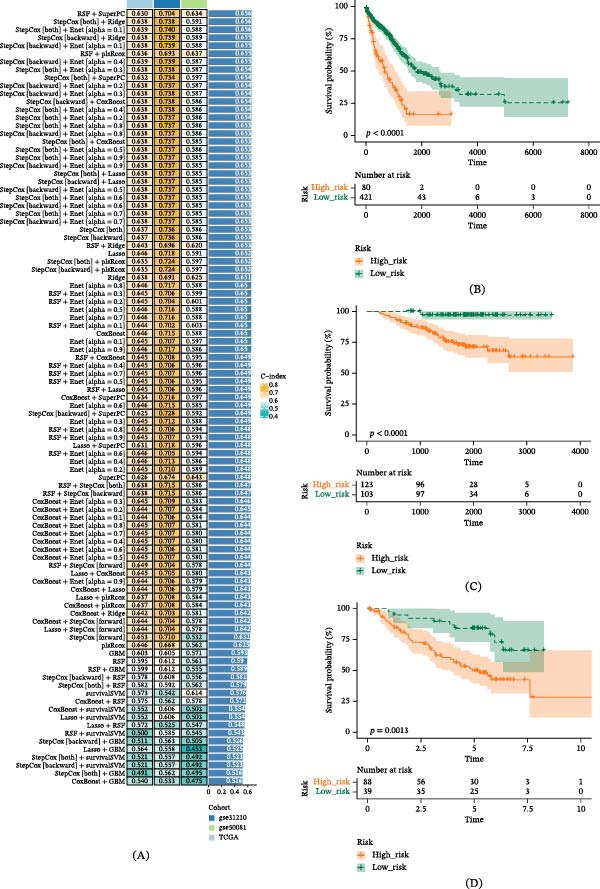
GABARF was devised and substantiated via an exhaustive program reliant on machine learning. (A) A total of 101 predictive models underwent screening via a 10‐fold cross‐validation framework. Subsequently, the C‐index for each model was computed across all validation datasets. (B–D) Utilizing the OS Kaplan–Meier curve of GABARF in the external validation set GSE31210/GSE50081 and TCGA training set, a log‐rank test was employed.

The construction of GABARF with stability and consistency plays a crucial role in analyzing clinical information and prognosis accuracy. We employ a combination of 101 machine learning algorithms to process the previously obtained 38 prognostic genes. Specifically, we utilize the TCGA‐LUAD dataset as the training set, fit 101 predictive models under the framework of 10‐fold cross‐validation, then compute the C‐index of all datasets. Among these 101 models, the RSF + SuperPC model not only has the highest average C‐index, but also performs excellently in the C‐index evaluation of the training set (TCGA) and two external validation sets (GSE31210 and GSE50081). In other words, this model is characterized by high accuracy and correlation (Figure 3A). Thus, based on the results of all C‐indexes, we preliminarily select the RSF + SuperPC model as the predictive model. Survival analysis data shows that the OS of patients in the low‐risk group from two validation sets and the training set is significantly higher than that of patients in the high‐risk group (Figure 3B–D).

### 3.4. Evaluation of the GABARF Model

The results of the ROC curve analysis indicate that in the training set, the AUC for GABARF at 1, 3, and 5‐year intervals are 0.64, 0.66, and 0.65, respectively. In the external validation set GSE31210, these values are 0.71, 0.68, and 0.73, respectively; while in the external validation set GSE50081, these values are 0.65, 0.64, and 0.68 (Figure 4A). This suggests that GABARF has the potential to become a reliable differentiation tool. Subsequently, we observed the TNM staging, gender, and survival status of the high‐risk and low‐risk groups in the training set. Our results show that compared to stage T1, the risk scores for stages T2 and T3 significantly increased (Figure 4B–F). These findings suggest that GABARF is associated with poor prognosis in LUAD patients. In fact, GABARF can predict the T‐stage of patients, with the diagnostic ROC curve showing a predicted AUC of 0.627 (Figure 4G), indicating that GABARF has the potential to predict the size and infiltration status of LUAD tissues. Lastly, the results of the Kaplan–Meier curve analysis demonstrate that GABARF has significant prognostic capability in the unfavorable subgroups of LUAD (Figure 4H).

Figure 4The evaluation of the GABARF model. (A) The ROC curve illustrates the specificity and sensitivity of GABARF in predicting 1, 3, and 5‐year overall survival (OS) within both the TCGA training set and the external validation set. (B) The correlation between the clinical features and the risk stratification (low and high) as predicted by GABARF. (C) The distribution of T staging within the risk subgroups as determined by GABARF. (D) Analysis of the distribution of clinical features and gene expression patterns in relation to the GABARF risk score. (E, F) The variation in risk scores among patients based on their T staging. (G) The ROC curve demonstrates GABARF predictive ability for LUAD metastasis. (H) The Kaplan–Meier curve illustrates the consistent performance of GABARF across different LUAD patient subgroups.  ^∗∗^ *p* < 0.01,  ^∗∗∗^ *p* < 0.001.

**Figure:**
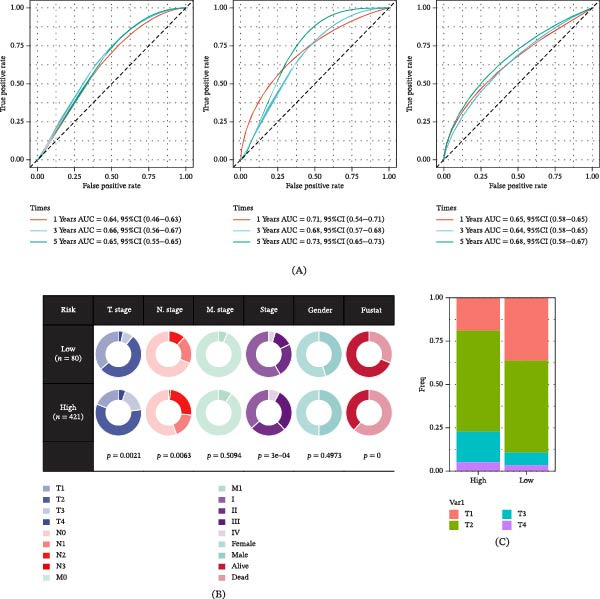

**Figure:**
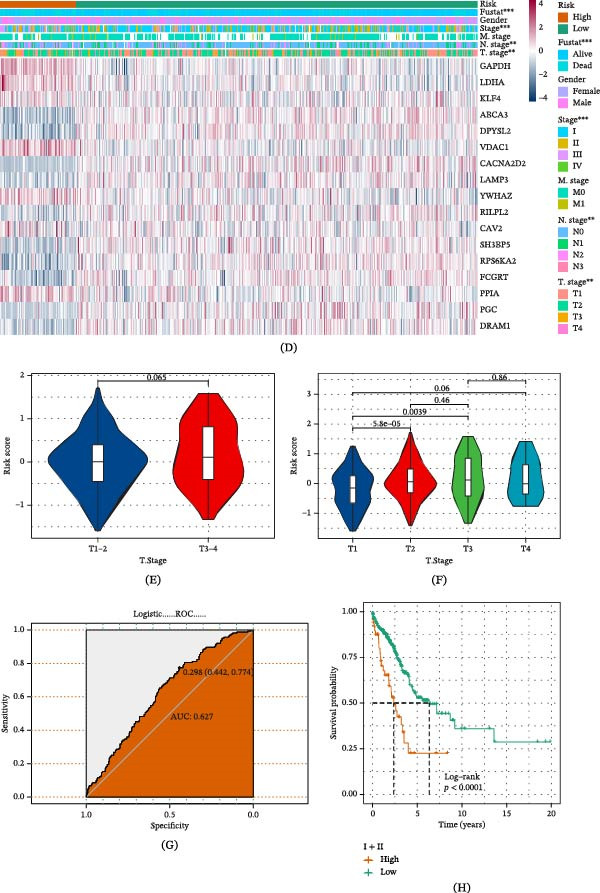

### 3.5. Nomogram Was Established and Its Performance Was Verified in Relation to Clinical Characteristics

**Figure 5:**
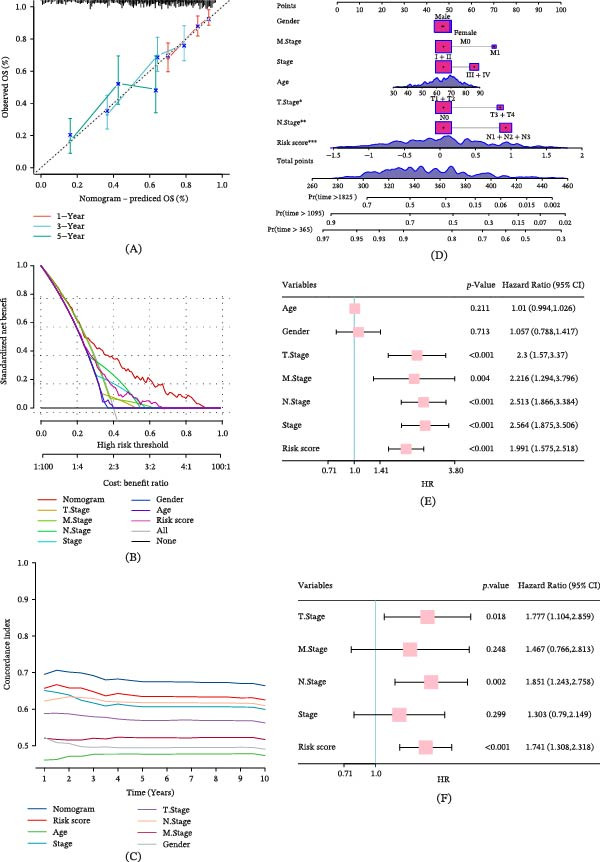
Establishment and validation of the nomogram. (A) Nomogram calibration curves for 1, 3, and 5‐year overall survival. (B) Decision curve analysis (DCA) illustrates the net benefit by applying the nomogram and other clinical features. (C) The C‐index in the figure is compared with other clinical characteristics. (D) The nomogram is built on the ICDRS and clinical characteristics, encompassing gender, age, grade, stage, and TNM staging. (E, F) Single and multiple variable analyses of clinical characteristics and GABARF in the overall survival of the TCGA‐LUAD cohort.  ^∗^ *p* < 0.05,  ^∗∗^ *p* < 0.01,  ^∗∗∗^ *p* < 0.001.

Our results demonstrate that in the univariate and multivariate Cox regression analysis of OS in the TCGA‐LUAD dataset, GABARF was identified as a risk factor for OS in the univariate analysis (Figure 5E). In the multivariate analysis, GABARF was still identified as an independent prognostic factor for OS (Figure 5F). These findings confirm that GABARF is an independent prognostic factor for LUAD, and GABARF possesses significant prognostic power for patients. The application of nomogram can enhance the clinical applicability of the GABARF evaluation system. First, we employed calibration curves to evaluate the consistency between nomogram predictions and actual data (Figure 5D), and the results showed good consistency in 1, 3, and 5‐year intervals, ensuring the prediction accuracy of the model (Figure 5A). Moreover, the C‐index analysis revealed that the nomogram model outperforms other clinical features in predicting OS within a 1–10‐year period (Figure 5C). Concurrently, the DCA demonstrated that the nomogram has a higher net clinical benefit compared to other clinical features (Figure 5B). These results suggest that the GABARF‐based nomogram has the potential to become a stable and reliable predictive model for personalized prognosis of LUAD.

### 3.6. Transcriptomics Suggested the Potential Molecular Interaction Mechanism of GABARF

Revealing the deep molecular characteristics and mechanisms accompanying the emergence of GABARF risk subgroups is the fundamental method for further studying the correlation between GABARF and LUAD prognosis. Based on the GO gene set, we performed GSEA analysis. The results showed that the low‐risk group was significantly enriched in KRAS signals, allograft rejection, inflammation, and metabolism, et cetera (Figure 6A), while the high‐risk group was significantly enriched in E2F targets, G2M checkpoint, MTORC1 signaling pathway, MYC targets, and glycolysis, et cetera (Figure 6B). Additionally, GSVA revealed that the low‐risk group exhibited enhanced activity in pathways related to heme metabolism, myogenesis, bile acid metabolism, coagulation, and NOTCH signaling (Figure 6C). The correlation analysis of these key pathways with GABARF further supports our finding that GABARF is closely linked to the immune and metabolic pathways related to tumors (Figure 6D). Next, we performed KM curve analysis on the Hallmark pathways to verify their association with LUAD prognosis. The results showed that the pathways enriched in the high‐risk group, such as E2F targets, G2M checkpoint, MTORC1 signaling pathway, MYC targets, et cetera, are all related to poor prognosis (Figure 6E–H). These results indicate that the changes in the aforementioned pathways may be one of the key factors leading to the prognostic differences in GABARF risk subgroups, and the common poor prognosis in the high‐risk group fundamentally stems from the abnormalities in the related signaling pathways.

Figure 6Transcriptional features of different patients in LUAD. (A) The ridge plot shows the richness of GO items in the low‐risk group. (B) GSEA analysis results show the richness of GO items in the high‐risk group. (C) The difference in the activity of marker pathways between high‐risk and low‐risk groups based on GSVA scores. (D) The correlation between risk scores and the activity of marker pathways based on GSVA scores. (E–H) Kaplan–Meier survival plots show a significant correlation between OS and GSVA scores for MYC checkpoint (F), MTORC1 signaling pathway (G), and G2M targets (H).

**Figure:**
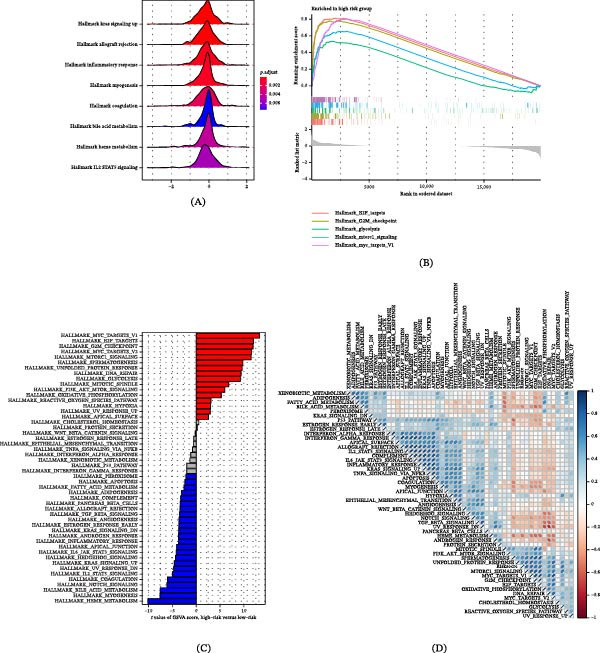

**Figure:**
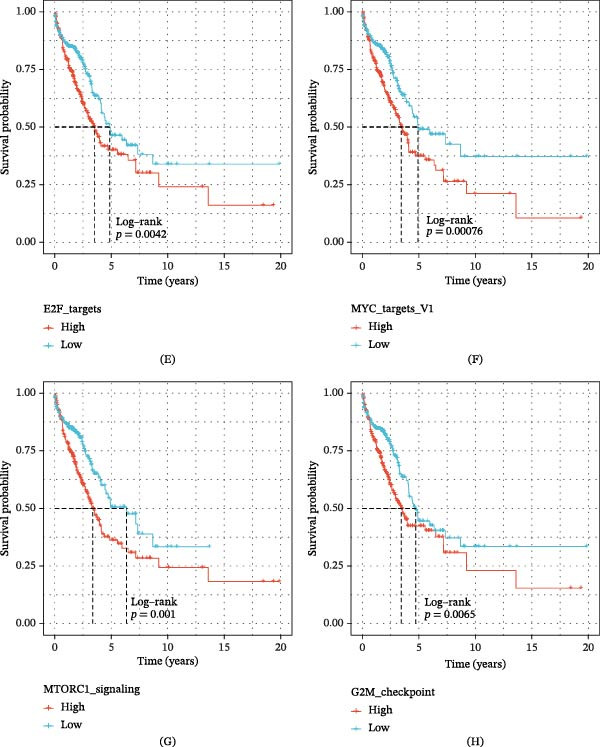

### 3.7. Intratumor Heterogeneity (ITH) and Genomic Variation in GABARF Risk Subgroups

ITH is characterized by the genetic alterations that occur due to numerous divisions and proliferation throughout tumor development, leading to variations in tumor growth rate, invasiveness, drug sensitivity, and prognosis. Generally, an elevated level of ITH is associated with increased tumor resistance and expedited cancer progression. ITH was quantified utilizing the MATH algorithm, indicating a direct correlation between higher MATH scores and increased ITH. Our study revealed that patients categorized in the high‐risk group of GABARF exhibited higher MATH scores compared to their counterparts in the low‐risk group (Figure 7A). Further investigation revealed a corresponding decrease in OS among patients with higher MATH scores (Figure 7B). Upon combining ITH and GABARF, it was observed that the prognosis was significantly less favorable for the “high‐risk plus high MATH score” group in comparison to the “low‐risk plus low MATH score” group (Figure 7C). Conversely, a significant mutation spectrum was identified between the GABARF high‐risk group and the low‐risk group, as illustrated in the mutation map (Figure 7D,E). For example, the mutation rate of TP53 gene in the high‐risk group was 70%, which was much higher than that in the low‐risk group (45%). TP53 is an important tumor suppressor gene, encoding p53 protein. Upon mutation, the gene loses its ability to respond to transcription or translation via the conventional p53 signaling pathway, leading to uncontrolled cell division and proliferation. Simultaneously, an analysis was conducted on the relationship between co‐occurring and exclusive mutations in the top 20 mutated genes within the two medium‐risk subgroups. The results indicated a higher frequency of co‐occurring mutations in the high‐risk group compared to the low‐risk group (Figure 7F,G). Additionally, the CNVs of 12 genes exhibited significant differences between the high and low‐risk groups. Specifically, FLG and CSMD3 primarily featured CNV gains, while COL11A1 predominantly exhibited CNV losses (Figure 7H).

Figure 7The figure presents gene alterations associated with GABARF in patients categorized as high or low risk. (A) A violin plot illustrates the disparity in mutant‐allele tumor heterogeneity (MATH) scores between groups of high and low risk. (B) The Kaplan–Meier curve depicts the differential in overall survival (OS) rates between high and low‐risk cohorts. (C) The Kaplan–Meier curve analysis of OS incorporates both MATH scores and GABARF risk scores. (D, E) The waterfall plot illustrates the somatic mutation landscape for high‐risk (D) and low‐risk (E) patients within the TCGA‐LUAD cohort. (F) A heatmap elucidates the relationship between co‐occurring and exclusive mutations of the top 20 mutated genes within high and low‐risk demographics. (G) The distribution of CNV frequency among DEGs is provided for high and low‐risk groups. In the context of this study, red and blue signify amplification and deletion, respectively, within cells assigned high‐risk and low‐risk scores.

**Figure:**
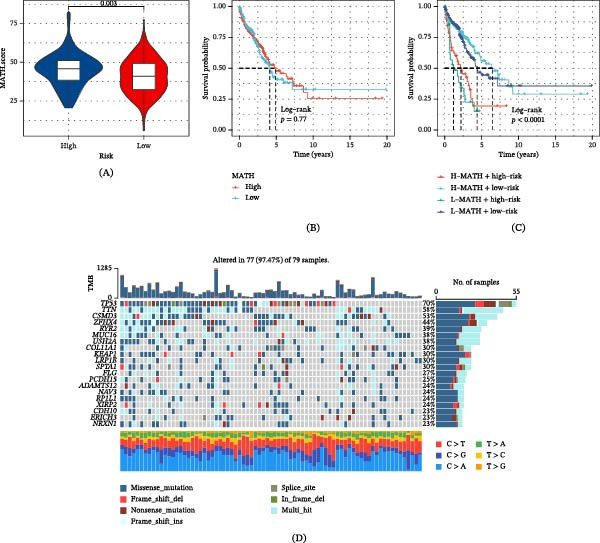

**Figure:**
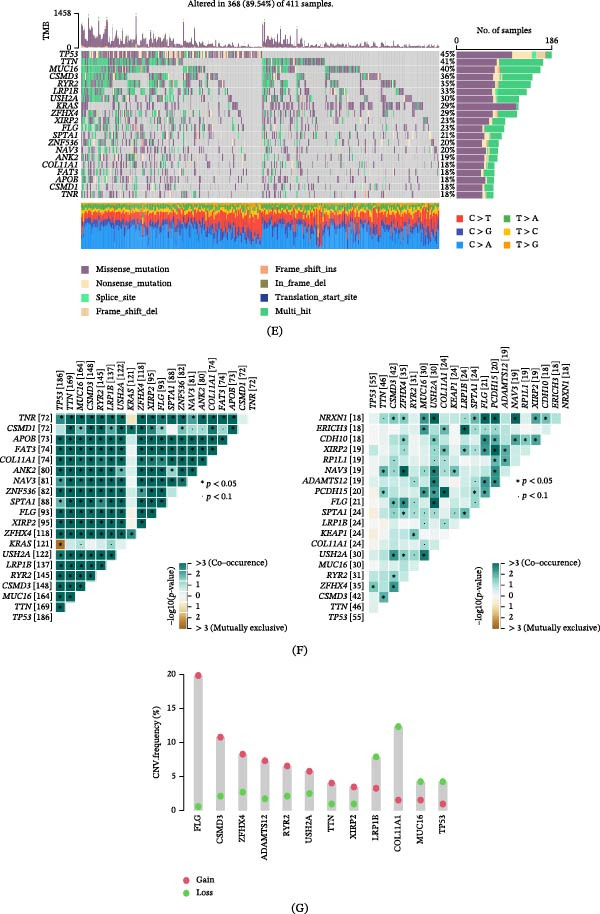

### 3.8. Correlation of GABARF With Single‐Cell Features

Given the heterogeneity among cells, it is necessary to analyze the relationship between GABARF at the single‐cell transcriptome level and the tumor microenvironment (TME). We studied the expression characteristics of GAPDH, LDHA, KLF4, ABCA3, DPYSL2, VDAC1, CACNA2D2, lysosome‐associated membrane protein 3 (LAMP3), YWHAZ, RILPL2, CAV2, SH3BP5, RPS6KA2, FCGRT, PPIA, PGC, and DRAM1 in various cell types. GAPDH, LDHA, ABCA3, CACNA2D2, LAMP3, PPIA, PGC, and DRAM1 are mainly expressed in immune cells, with macrophages being the majority (Figure 8A). We attempted to categorize the various cells in the TME, especially tumor epithelial cells, into high‐risk and low‐risk groups and analyze their interactions. The results showed significant differences in communication patterns between cells classified as high‐risk GABARF group and those classified as low‐risk group (Figure 8B–E). Various cells in the TME played roles as initiators, receptors, mediators, or participants in cell signal transduction, thereby establishing signal transmission links between cells. In the TME, epithelial cells with higher risk scores tended to establish connections with more types of cells in the migration inhibitory factor (MIF) signaling pathway and play important roles, while epithelial cells with lower risk scores were more likely to establish connections with other cells in the APP pathway (Figure 9A–E). In other words, these cells may regulate the adhesion, differentiation, and migration of tumor cells, and have an impact on the survival of cancer cells.

Figure 8The study examines the correlation between GABARF and the characteristics of single cells. (A) The expression of multiple genes across various cell types is analyzed using single‐cell RNA sequencing. (B) Differential gene expressions between high‐risk and low‐risk cells are analyzed using the KEGG pathway. (C) The enrichment of Gene Ontology (GO) terms in low‐risk cells is identified through gene set enrichment Analysis (GSEA). (D) The study investigates the interactions between ligands and receptors released from high‐risk cancer cells. (E) The interactions between ligands and receptors originating from low‐risk cancer cells are examined.

**Figure:**
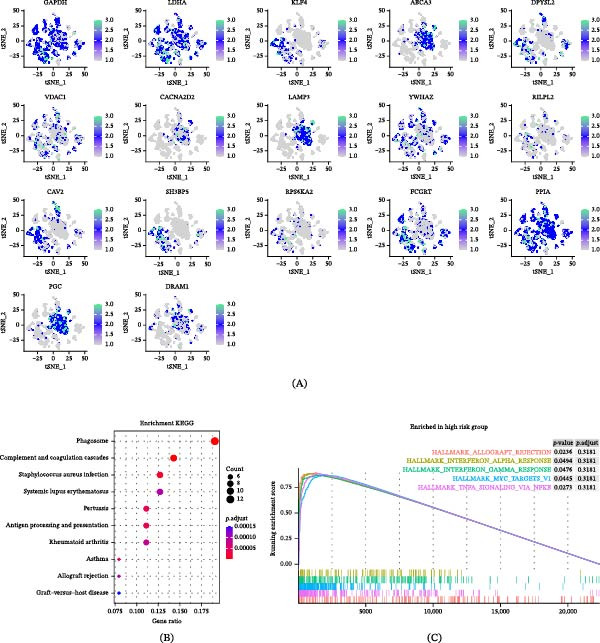

**Figure:**
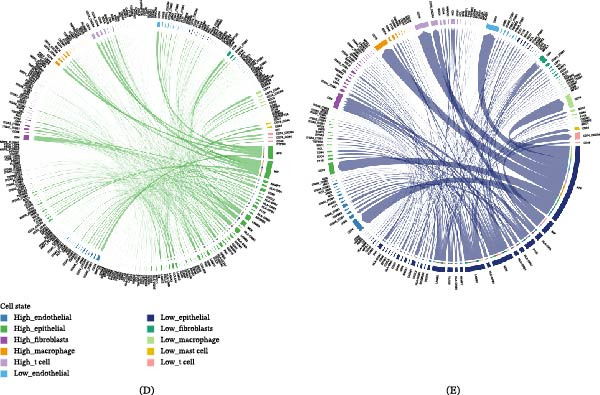

Figure 9The correlation between GABARF and single‐cell characteristics is demonstrated. (A–F) The Circos diagram displays the signaling pathway networks of APP (A, D), MIF (B, E), and MK (C, F), while the heatmap shows the role of different cell types in the pathway network.

**Figure:**
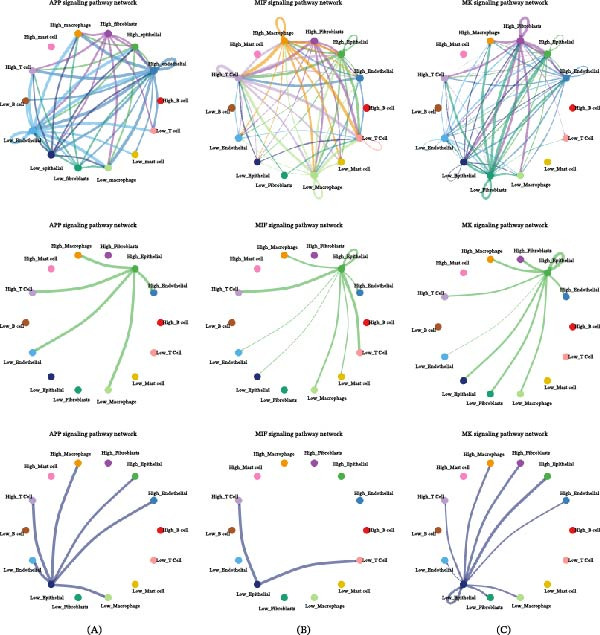

**Figure:**
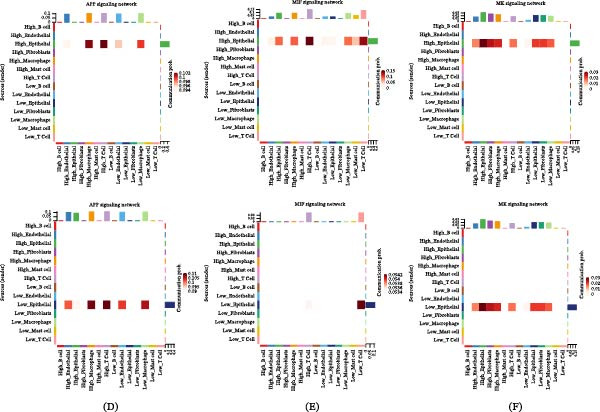

### 3.9. Association of GABARF With Characteristics of TIME

**Figure 10:**
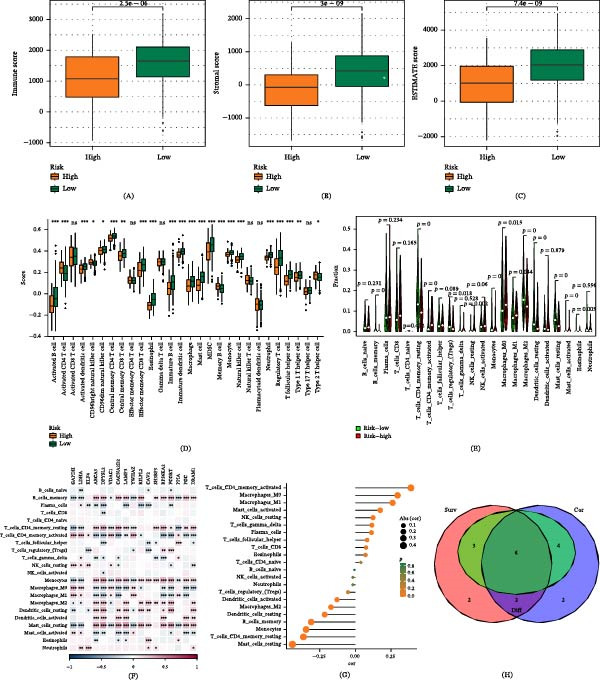
The immune features associated with GABARF in LUAD are examined. (A–C) The immune score (A), matrix score (B), and ESTIMATE score (C) are utilized to quantify the differences in immune status between high‐risk and low‐risk groups. (D, E) Quantifying the abundance of various TME infiltrating cell types between high‐risk and low‐risk groups via the ssGSEA algorithm (D) and CIBESORT algorithm (E). (F) The relationship between TME infiltrating cells and the inherent GABARF gene is explored. (G) Correlation analysis between TME infiltrating cells and GABARF is performed. (H) A Venn diagram is used to show the differential analysis, correlation analysis, and survival analysis of intersecting TME infiltrating cell types.  ^∗^ *p* < 0.05,  ^∗∗^ *p* < 0.01,  ^∗∗∗^ *p* < 0.001.

The immune infiltration status of LUAD samples was assessed using the ESTIMATE algorithm, which was utilized to calculate the immune score, matrix score, and ESTIMATE score for risk subgroups. The low‐risk group exhibited higher scores across these three evaluations (Figure 10A–C). The CIBERSORT algorithm was employed to further delineate the differences in specific immune cell infiltration between the high‐risk and low‐risk groups. This was validated with concordant results obtained through the ssGSEA and Xcell algorithms. The results indicated that type 1 helper cells, T follicular helper cells, and activated dendritic cells (DCs) were more abundant in the low‐risk group, while resting NK cells, type 2 helper cells, memory B cells, and monocytes were more abundant in the high‐risk group (Figure 10D). Our findings revealed that within GABARF, 17 genes exhibited a high correlation with tumor‐infiltrating immune cells. Among them, genes such as ABCA3, DPYSL2, and CACNA2D2 are positively correlated with memory B cells, while SH3BP5 and FCGRT genes are positively correlated with monocytes (Figure 10E). Simultaneously, we identified 22 cell types significantly correlated with GABARF through Spearman correlation analysis (Figure 10F). Finally, by integrating the results of the three analyses, an intersection comprising six types of TME cells was identified, which was visualized using an overlapping Venn diagram (Figure 10G). These results demonstrate that these six types of immune cell infiltration play a vital role in the onset, development, and prognosis of LUAD.

### 3.10. The Effect of Immunotherapy and the Relationship Between Anti‐Tumor Immune Circulation and GABARF

**Figure 11:**
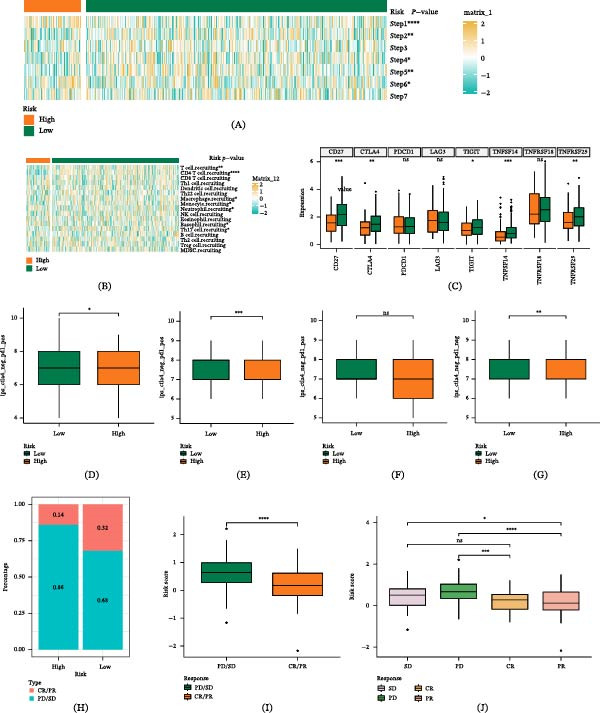
This study explores the correlation between GABARF and the effectiveness of immunotherapy or anti‐tumor immunity. (A) A heatmap illustrates the disparities in the seven‐step activities of the cancer immunity cycle between high‐risk and low‐risk groups. (B) A heatmap demonstrates the disparities in the ability of GABARF subgroups to recruit immune cells. (C) The study examines the expression of immune checkpoints in high‐risk and low‐risk populations. (D–G) The study presents the IPS scores for the application of four distinct combinations of immune checkpoint inhibitors in high‐risk and low‐risk groups. (H) The study calculates the proportion of PD/SD or CR/PR patients receiving immunotherapy within the high‐risk and low‐risk groups of the IMvigor210 cohort. (I) A box plot depicts the difference in risk scores between CR/PR patients and SD/PD patients within the IMvigor210 cohort. (J) A box plot reveals the fluctuations in risk scores among SD, PD, CR, and PR patients within the IMvigor210 cohort.  ^∗^ *p* < 0.05;  ^∗∗^ *p* < 0.01;  ^∗∗∗^ *p* < 0.001,  ^∗∗∗∗^ *p* < 0.0001.

Tumor cells exist in a complex immune microenvironment, and merely analyzing immune cell infiltration does not provide a comprehensive and in‐depth understanding of immune activation and exhaustion. We focus on the process of anti‐tumor immunity, comprehensively evaluate the effectiveness of immune treatment, and deeply depict the anti‐cancer role of immune cells. Our research results show that compared with the high‐risk group of GABARF, the low‐risk group shows stronger activity in steps 1, 2, 4, 5, and 6 (Figure 11A). We further analyzed the recruitment of different immune cells in different risk subgroups in step 4. The results show that the low‐risk group has stronger abilities to recruit T cells, CD4^+^ T cells, macrophages, and monocytes (Figure 11B). These results suggest that the low‐risk group has stronger anti‐cancer activity in immune cell circulation. Existing studies point out that high expression of immune checkpoints can enhance the therapeutic effects of ICIs. We have compiled the comparative data of immune checkpoint expression between the GABARF risk subgroups we analyzed. All the immune checkpoints we detected showed significantly higher expression characteristics in the low‐risk group, such as the expression levels of CD27, CTLA‐4, TIGIT, TNFSF14, and TNFRSF25 (Figure 11C). As higher IPS scores usually indicate better ICI treatment effects, we planned combinations of four typical ICI treatment drugs (PD‐1 inhibitors and CTLA‐4 inhibitors) to compare the IPS scores of each group: CTLA‐4(−) PD‐1(−) treatment, CTLA‐4(−) PD‐1(+) treatment, CTLA‐4(+) PD‐1(−) treatment, and CTLA‐4(+) PD‐1(+) treatment. The results show that the IPS of anti‐CTLA‐4 treatment and combined anti‐CTLA‐4 and anti‐PD‐1 treatment in the low‐risk group is significantly higher, and the response of patients in the low‐risk group to treatment is better than that of the high‐risk group (Figure 11D–G). We further validate the adaptability of GABARF to patients’ immune treatment by choosing to include the IMvigor210 cohort receiving atezolizumab treatment. Using the GABARF model, we calculate the risk scores of patients in this cohort and divide them into high‐risk and low‐risk groups. As shown in the Figure 11, after the chi‐square test, the complete remission/partial remission (CR/PR) ratio of the high‐risk group is significantly lower than that of the low‐risk group, and the progressive disease/stable disease (PD/SD) ratio of the high‐risk group is significantly higher than that of the low‐risk group (Figure 11H). In addition, the risk scores of PD/SD patients are significantly higher than those of CR/PR patients (Figure 11I,J). These findings suggest that GABARF has the potential to predict the effectiveness of immunotherapy, indicating that patients in the low‐risk group might derive greater benefit from this treatment.

### 3.11. Correlation Analysis of Drug Sensitivity of GABARF

**Figure 12:**
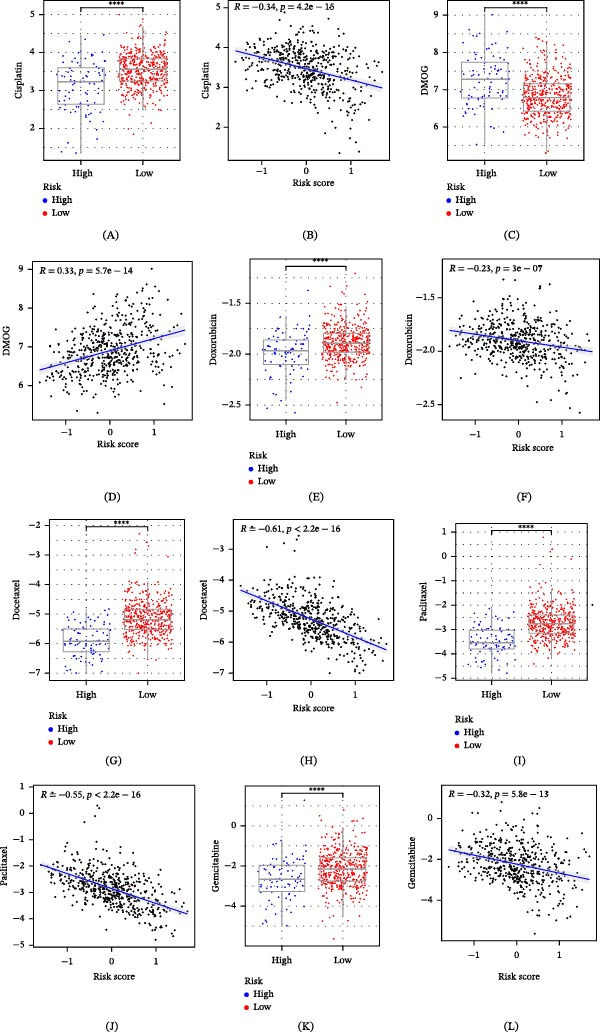
The relationship between GABARF and drug sensitivity. (A, C, E, G, I, K). Comparison of drug sensitivity between high‐risk and low‐risk groups to first‐line LUAD drugs, including Cisplatin, Doxorubicin, Paclitaxel, DMOG, Docetaxel, and Gemcitabine. (B, D, F, H, J, L). The correlation between the LUAD risk score and IC50 of small molecule drugs such as Cisplatin, Doxorubicin, Paclitaxel, DMOG, Docetaxel, and Gemcitabine.  ^∗∗∗∗^ *p* < 0.0001.

In the treatment regimens for non‐small cell LUAD, standard first‐line chemotherapy drugs are extensively applied. However, the situation of chemotherapy resistance frequently arises due to the highly heterogeneous and volatile TME. Consequently, we investigated the susceptibility of GABARF to six primary chemotherapy drugs, namely Cisplatin, Doxorubicin, Paclitaxel, DMOG, Docetaxel, and Gemcitabine. The sensitivity to these drugs was assessed through the half‐maximal inhibitory concentration (IC50 value). The findings revealed that, in comparison to the low‐risk group, the IC50 values for five drugs, excluding DMOG (Figure 12D), were significantly lower in the high‐risk group (Figure 12A–C, E,F), suggesting the high‐risk group has a greater sensitivity to the majority of standard chemotherapy drugs. Concurrently, the GABARF risk score displayed a positive relationship with the IC50 value of DMOG, while it was negatively correlated with the IC50 values of the other five drugs (cisplatin, doxorubicin, paclitaxel, docetaxel, and gemcitabine). These findings imply that patients in the low‐risk group might exhibit a stronger reactivity to DMOG and immunotherapeutic methods, whereas patients in the high‐risk group might have an increased sensitivity to standard chemotherapy drugs.

### 3.12. Impact of LDHA Overexpression on Cancer Cell Proliferation and Apoptosis

To identify the most promising candidate gene for functional validation, we collected 10 clinical samples for analysis. Given that our predictive model comprised 38 genes in total, which precluded comprehensive experimental validation of all candidates, we strategically selected four key genes for qPCR analysis: the two genes with the highest risk scores (LDHA and VDAC1) and the two genes showing the most pronounced changes in CNV analysis (YWHAZ and EIF4A3) (Figure S1). qPCR results demonstrated that LDHA exhibited the most significant differential expression among these four genes, warranting further functional investigation.

**Figure 13:**
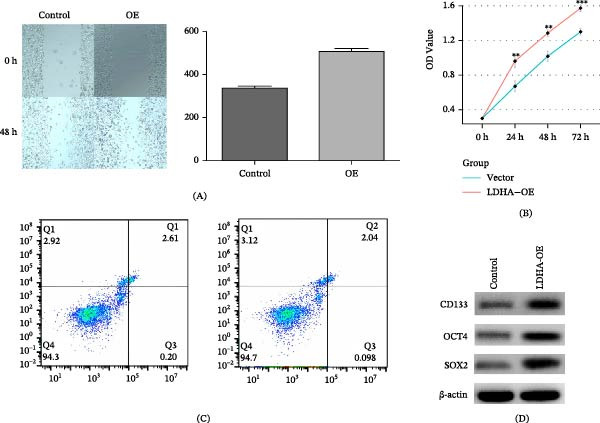
LDHA affects tumor proliferation and apoptosis. (A) LDHA overexpression results in increased tumor cell proliferation. (B) LDHA overexpression results in CCK8 assay. (C) LDHA overexpression is associated with reduced apoptosis in tumor cells. (D) Representative western blot images showing the expression of tumor stemness markers (CD133, SOX2, and OCT4) in control and LDHA‐overexpressing LUAD cells.

Subsequently, we conducted a series of functional validation experiments on LDHA. In scratch assays, cells overexpressing LDHA demonstrated notable acceleration in wound healing, with substantially increased migration speed compared to control groups across three independent trials, indicating LDHA’s pivotal role in promoting cell migration (Figure 13A). Furthermore, CCK8 assays revealed enhanced cell proliferation capacity in LDHA‐overexpressing cells, with significantly increased cell viability relative to control groups, demonstrating the gene’s contribution to promoting cell proliferation (Figure 13B). Additionally, apoptosis assays demonstrated a marked reduction in cell death among LDHA‐overexpressing cells, highlighting the gene’s critical role in enhancing cell survival (Figure 13C). To further evaluate the effect of LDHA on tumor cell stemness, we overexpressed LDHA in LUAD cells and performed Western blot analysis to detect the expression of stemness‐associated markers, including CD133, SOX2, and OCT4. As shown in Figure 13D, LDHA overexpression markedly increased the protein expression levels of these stemness markers, suggesting that LDHA may enhance the stem‐like properties of tumor cells.

## 4. Discussion

The anomalous aggregation of GABA in particular neoplastic tissues has been delineated in initial immunohistochemical investigations. However, the implications of GABA‐associated genes and transcriptomic characteristics in disease prognosis, and the molecular–immunological interplay mechanism of GABA in tumor occurrence and development, remain elusive. LUAD is a prevalent malignant respiratory neoplasm, with elevated incidence and mortality rates. Despite considerable investigation into lung cancer biomarkers in recent years, no sensitive and dependable markers for LUAD have been identified. As per scholarly reports, the concentration of GABA in lung cancer tissues has markedly escalated [8], which lays the groundwork for the development of a comprehensive and specific clinical prognostic model for GABA. We have pioneered a novel algorithmic framework, incorporating 10 machine learning methodologies and their 101 permutations, to construct the GABARF system. Not only can GABARF efficaciously and precisely prognosticate the clinical outcome of LUAD, it can also stratify the risk of LUAD patients and assess their potential benefits from either immunotherapy or standard first‐line chemotherapy. We have not only corroborated the correlation between GABARF and the progression and prognosis of LUAD, but also executed multiomics analyses encompassing genomics and transcriptomics, uncovering the molecular underpinnings and potential immunological mechanisms of GABARF in tumorigenesis and progression. We employed three methodologies, namely AddModuleScore, ssGSEA, and WGANA, to explore GABA‐associated genes that could potentially serve as therapeutic targets for LUAD. Our discoveries furnish biological substantiation and theoretical underpinnings for the application of GABARF in directing clinical decisions in immunotherapy or chemotherapy, and also offer practical multi‐omics data and theoretical framework for future GABA‐oriented research in LUAD.

GABA primarily exercises its function through the binding to chloride‐directed GABAARs and G‐protein‐coupled GABAB receptors. The prevailing understanding is that GABA signaling has a pivotal role in cell proliferation, differentiation, and migration. However, the regulatory role of the ubiquitously expressed GABA and GABAAR in cellular signaling activities (excluding neurotransmitter signal transduction) across various organs and tissues, including the lungs [15], liver [16], and pancreas [17], remains insufficiently explored. The rapid proliferation of tumorous cells is contingent upon the depolarization of their membrane potential [18]. In comparison to normal tissues, anomalous alterations in ion channels could potentially foster the initiation and progression of tumors [19]. As reported, cancer cells exhibiting high GABAAR expression during proliferation present an electrophysiological state analogous to GABAAR‐related signal activities observed in neural or embryonic development [20]. Notably, the overexpression of GABA and GABAAR‐related genes correlates with a poor prognosis in patients with non‐small cell lung cancer (NSCLC) [21]. Accumulating evidence suggests that the expression patterns of GABA and its receptors in tumors could potentially reflect tumor proliferation or migration status and also guide the evaluation of patient prognosis, involving intricate molecular and immune mechanisms. However, comprehensive and systematic research on GABA‐related mechanisms in LUAD is currently lacking. The aim of this study is to initially analyze and summarize GABA‐related molecules and immune mechanisms in LUAD from a multiomics perspective. According to the study’s findings, we observed that all genes encompassed in the GABARF influence tumor development or partake in associated immune response activities. For instance, YWHAZ, primarily secreted by cells like M2 macrophages and Tregs, can inhibit pro‐inflammatory factors [22] and is significantly upregulated in lung cancer, participating in various processes, including cell proliferation, invasion, and migration [23, 24]. Eukaryotic initiation factor 4 A‐III (elF4A3), serving as the central helicase portion of the exon junction complex, exhibits a higher level in cancer compared to normal tissues. The inhibition or reduction of its encoded protein may mediate the cessation and death of cancer cell division [25]. CD63, alternatively known as LAMP3, is associated with exosomes that express LAMP3, enhancing the antigen presentation process to activate CD4^+^ T cells and thereby modulating tumor immune activities [26]. LDHA‐mediated glycolysis plays a crucial role in the proliferation, differentiation, and activation of B cells. Even a deficiency of LDHA in T cells can impair the clonal expansion of activated B cells [27]. These findings indirectly substantiate that GABARgenes hold potential for development as predictive tools reflecting the tumor characteristics or immune features of LUAD patients. Mechanistically, the link between LDHA and GABAergic signaling in LUAD is driven by metabolic reprogramming. GABA is synthesized from glutamate. LDHA overexpression accelerates glycolysis, forcing cancer cells to heavily rely on glutaminolysis, which depletes the intracellular glutamate pool and thereby directly couples glucose metabolism with GABA synthesis. Furthermore, LDHA‐driven lactate accumulation acidifies the TME, which can modulate GABA receptor (GABAR) sensitivity and synergize with GABA to strongly inhibit anti‐tumor immunity.

This study identifies GABARF as a high‐precision biomarker with broad applicability for reflecting the clinical prognosis of LUAD. GABARF was constructed using a framework that encompasses 101 combinations of 10 machine learning algorithms. The framework simplifies the model via data dimensionality reduction. Subsequently, Kaplan–Meier curve analysis was utilized, establishing that GABARF can conduct risk assessment and grading for LUAD patients, based on OS. Additionally, GABARF can serve as an independent predictive factor. A significant correlation was found between the high‐risk group of GABARF and the adverse clinical outcomes of LUAD. Importantly, a nomogram model was constructed that incorporates GABARF and other clinical features, demonstrating good discriminatory power in quantifying a patient’s survival status. This nomogram model, compared to other clinical features, offers a higher clinical net benefit to patients, signifying its conciseness and reliability as a tool for predicting LUAD patients’ survival time. Overall, this is the pioneering study exploring and validating the potential application of GABA as an individualized indicator for treatment response and prognosis in LUAD. Accordingly, a large‐scale machine learning framework was developed that integrates genomics and transcriptomics, establishing GABARF with high predictive accuracy and clinical application value. Based on these features, the developed nomogram model can effectively and accurately quantify LUAD patients’ expected survival risk. Our findings suggest that GABA holds significant potential for predicting the efficacy and prognosis of LUAD patients. The comprehensive application of GABA not only aids in improving clinical outcomes but also offers guidance in formulating treatment plans and determining treatment depth.

By leveraging multiomics technologies, we can probe into the molecular mechanisms of LUAD at multiple levels and identify new biomarkers by recognizing DNA, RNA, proteins, and other metabolites. We investigated the interaction between the biological functions and signaling pathways of the GABARF risk subgroups, and found that in the low‐risk group, inflammatory responses and metabolic activation processes showed a trend of enrichment, which could account for the better prognosis observed in the low‐risk group. Conversely, we observed a significant enrichment of signaling pathways, such as E2F, MYC, and MTORC1, in the high‐risk group. E2F is a crucial transcription factor that plays a significant role in maintaining normal cellular homeostasis. However, when its transcription target is dysregulated, its expression activity increases, resulting in uncontrolled cell proliferation and promoting the occurrence and progression of cancer [28]. Similarly, the MYC transcription factor is a common somatic mutation oncogene, and its activity has reached a high level in primary early tumors. These tumors often exhibit transcriptional features associated with poor prognosis, which may mean that MYC indirectly drives late‐stage tumor metastasis to some extent [29, 30]. Notably, we observed an upregulation of E2F and MYC activity in T‐cell lymphoma, which may mediate tumor immune escape [31]. MTORC1 plays a crucial role in regulating protein synthesis and cell growth, acting as an essential signal for cell proliferation [32]. In cancer cells, excessive cell proliferation caused by genetic mutations or epigenetic changes is common, and currently, MTORC1 is considered one of the most important regulatory factors in cancer [33]. Research has reported that by downregulating MTORC1, the proliferation, migration, and invasion of LUAD tumor cells can be inhibited [34]. Furthermore, suppressing the expression of MTORC1 in monocytes and DCs can exert pro‐inflammatory effects [35], and MTORC1‐guided translation of IL‐10 takes precedence over IL‐12 expression [36]. These characteristics of GABARF‐related cancer‐promoting factors or pathways, associated with poor clinical prognosis, further substantiate the poor prognosis in the high‐risk group. Targeted drugs specifically designed for these aberrantly activated factors or pathways may potentiate anti‐tumor immunity, helping to inhibit the progression of LUAD. On the other hand, we noted a significant increase in the ITH of the GABARF high‐risk group compared to the low‐risk group. Combining our previous observations that patients in the high‐risk group have a more pronounced tendency to metastasize, we infer that GABARF has potential in predicting tumor metastasis. Our findings align with other studies indicating that tumors with higher ITH are more susceptible to metastasis and invasion [37]. In the high‐risk group, the mutation frequency of the key tumor suppressor gene TP53 is higher. TP53 is the most frequently mutated gene in human cancers, and its mutation is related to anti‐tumor immune suppression, often appearing together with poor prognosis of tumors such as LUAD [38–40]. These results highlight the characteristics of GABARF high‐risk group patients, including a low response to immunotherapy and poor prognosis.

The GABARF evaluation system, based on single‐cell transcriptome sequencing, includes genes that are significantly expressed in macrophages, endothelial cells, and T cells. This implies that these genes could potentially regulate tumor cell activity by influencing the TME through immune cells or paracancerous cells. The characteristic genes and signaling pathways implicated in tumor progression and immune processes, as summarized and analyzed at the single‐cell transcriptome level, align with the results from extensive transcriptomic/genomic analyses. These characteristic factors exhibit similar trends in the differences observed between GABARF risk subgroups obtained from two analytical perspectives. Furthermore, our findings indicate that tumor epithelial cells with higher GABARF risk scores have a more significant role in the macrophage MIF signaling pathway. MIF is a cytokine that plays a key role in immune and inflammatory responses, and can increase the risk of various cancers, including lung cancer. The MIF signaling pathway, often upregulated in epithelial cells and LUAD, is a crucial pathway [41]. The upregulated MIF in turn counteracts type II alveolar epithelial cells, inducing morphological changes and promoting their abnormal proliferation, migration, and invasion [42]. Tumor epithelial cells with high GABARF scores may regulate the function of immune cells in the TME, or affect the death, proliferation, and other life activities of related cells, by intervening in pathways such as the MIF signal. These findings further propose GABARF as a prognostic indicator associated with tumor progression and immune processes.

The effectiveness of tumor immunotherapy primarily depends on the TME, specifically, the TIME [43]. TIME consists of various elements, including Th1 cells and NK cells. These are pro‐inflammatory cells that induce cytotoxicity in tumor cells via different mechanisms. DCs, known for their potent antigen‐presenting abilities, play a critical role in maintaining adaptive immunity in TIME. Moreover, TIME includes immune‐suppressive cells such as Th2 cells and certain B cell subsets [43, 44]. Abnormal expression of GAD1 in lung cancer leads to increased GABA synthesis. This stimulates the activation of GABAB receptors, enhances the β‐catenin signal, and significantly promotes continuous tumor cell proliferation [8]. Silencing GAD1 results in a significant increase in immune cell infiltration, including Th1 cells, CD8^+^ T cells, and CD103^+^ DCs. CD103^+^ DCs act as mediators for GABA secreted by the microenvironment, thereby recruiting T cells. CD103^+^ intraepithelial DCs are present in lung tissues, where they extend their dendrites between epithelial cells to acquire antigens [8]. When released by B cells, GABA can directly suppress the functionality of CD8^+^ T cells and stimulate the differentiation of monocytes towards an anti‐inflammatory phenotype, thereby undermining anti‐tumor immunity [13]. While our current study profoundly highlights the impact of GABARF on the immune microenvironment, it is critical to acknowledge the cellular origin of GABA within LUAD. Existing literature solidly establishes that malignant epithelial (tumor) cells, characterized by high expression of GABA‐synthesizing enzymes (GAD1 and GAD2), serve as the primary producers of GABA, whereas immune cells typically act as responders. This spatial division of labor reveals a complex bidirectional signaling axis between tumor cells and infiltrating immune cells. Specifically, tumor‐derived GABA functions as a critical paracrine signaling molecule; upon secretion, it binds to specific GABARs on various immune cells, inducing an immunosuppressive phenotype (e.g., macrophage polarization and CD8^+^ T cell inhibition). In a reciprocal manner, these reprogrammed, immunosuppressive immune cells secrete distinct cytokines and chemokines that feed back to the tumor cells, thereby reinforcing tumor stemness, immune evasion, and metabolic adaptation. Therefore, the prognostic and immunotherapeutic predictive value of our GABARF model fundamentally captures this dynamic, bidirectional neural‐immune crosstalk, rather than simply measuring autonomous immune cell activity. Consistent with this bidirectional crosstalk, our research shows that the GABARF high‐risk group has a relatively low level of immune infiltration, primarily consisting of immune‐suppressive or unactivated cells such as Th2 cells and resting NK cells. Conversely, we observed a high degree of immune infiltration in the low‐risk group, characterized by Th1 cells, activated DCs, et cetera, along with high activity in most phases of the anti‐cancer immune cycle. The GABARF low‐risk group demonstrates stronger anti‐tumor immune activity, a finding supported by the functional enrichment analysis. While earlier studies established that GABA promotes macrophage M2 polarization and suppresses CD8^+^ T cells through isolated mechanisms like NF‐κB/STAT3 inhibition, our GABARF signature specifically supersedes these findings by shifting from a single‐pathway paradigm to a system‐level network. Beyond localized immune suppression, GABARF captures profound tumor‐intrinsic metabolic shifts (e.g., enhanced glycolysis) and stemness enhancements (e.g., E2F targets). Furthermore, our single‐cell analysis uncovers novel intercellular crosstalk, showing that GABARF high‐risk epithelial cells actively orchestrate the microenvironment via MIF and APP signaling. Thus, GABARF advances beyond basic mechanistic models by translating this holistic neural‐immune‐metabolic landscape into a quantifiable clinical prognostic tool.

Emerging evidence suggests that GABA signaling may regulate cellular stemness through β‐catenin/Wnt activation [10]. The GABARF high‐risk group exhibited features associated with cancer stem cells: higher tumor heterogeneity, reduced immune infiltration, and elevated expression of proliferation pathways (E2F, MYC, and MTORC1). These observations suggest GABA‐mediated programs promote stemness maintenance in LUAD cells. GABA‐induced immunosuppression may create a microenvironment favorable for stem‐like tumor cell self‐renewal, as immunosuppressive conditions often correlate with increased stemness in cancers.

Currently, ICIs therapy is a well‐established method in clinical immunotherapy [45, 46]. Despite its significant role in extending the survival period of cancer patients, the therapy still faces the challenge of low response rates, regardless of whether it is used as a standalone treatment or in combination with conventional chemotherapy drugs. Our research demonstrates that GABARF can potentially predict the sensitivity of LUAD patients to ICI treatment. The low‐risk group, as classified by GABARF, exhibits an increased expression of immune checkpoint genes, and our IPS analysis identified that patients within the low‐risk group are more responsive to anti‐PD‐1 or combined anti‐CTLA‐4 and anti‐PD‐1 treatment. Several studies suggest that targeting GABA signals alongside immune checkpoint blockers (ICBs) could enhance the outcomes of immunotherapy for patients with various types of cancer [8, 13, 47, 48]. Inhibiting GABA production can work synergistically with ICBs to overcome the resistance of tumors to immunotherapeutic drugs [8, 47]. These research findings indirectly corroborate our results. Additionally, we confirmed the predictive capability of GABARF with respect to immunotherapy responses from another perspective. Using the IMvigor210 dataset, we assessed the ratios of CR/PR to PD/SD across different risk subgroups of patients. We found that the CR/PR ratio in the high‐risk group is significantly lower than that in the low‐risk group, while the PD/SD ratio is notably higher. Therefore, we can assert that GABARF holds substantial predictive value for immunotherapy outcomes, with patients in the low‐risk group, as classified by GABARF, being more likely to benefit from it. Furthermore, we conducted a sensitivity analysis of standard LUAD chemotherapy drugs across various GABARF risk subgroups. We found that the high‐risk subgroup displays greater sensitivity to most conventional chemotherapy drugs than the low‐risk group. We found that the high‐risk subgroup displays greater sensitivity (lower IC50) to conventional chemotherapy drugs. Mechanistically, this is driven by the hyperactive cell cycle state of high‐risk tumors; our GSEA results showed significant enrichment of E2F targets and G2/M checkpoints, rendering these rapidly dividing cells inherently more susceptible to DNA‐damaging and anti‐mitotic chemotherapeutics. Based on these findings, we deduce that patients in the high‐risk group are better suited for treatment with conventional chemotherapy drugs, while those in the low‐risk group may exhibit greater reactivity to immunotherapy strategies. Considering the vital role of GABA in antitumor immunity, therapeutically targeting GABA could potentially develop into a novel anticancer treatment strategy. Currently, innovative antitumor medications targeting GABA have not been developed in the preclinical stage. Through a comprehensive bioinformatics analysis of multiomics data, our study identified GABA‐associated genes that could serve as potential therapeutic targets for GABA in LUAD.

Importantly, our drug sensitivity analyses revealed an unexpected interaction that could significantly inform drug repurposing strategies. While the high‐risk group demonstrated heightened sensitivity to conventional chemotherapeutics (e.g., cisplatin and paclitaxel), the low‐risk group showed an unexpected, significant sensitivity to DMOG (dimethyloxalylglycine), a competitive inhibitor of HIF prolyl hydroxylase. Although DMOG is not a standard first‐line agent for LUAD, its potential to modulate hypoxic metabolism and immune cell metabolic reprogramming aligns perfectly with the enriched metabolic and inflammatory signatures observed in our low‐risk group. This finding suggests a highly promising repurposing strategy: deploying DMOG as a metabolic modulator to further synergize with or enhance the efficacy of ICIs in low‐risk GABARF patients.

Although GABARF demonstrates promising prognostic performance, its clinical implementation requires overcoming several technical barriers. GABARF is based on RNA‐seq data, which demands rigorous sample management and sequencing platform standardization. The variation in AUC across external validation sets (0.64–0.73) suggests the model performance may be suboptimal in certain patient populations, necessitating further validation in larger and more diverse cohorts (≥500–1000 patients) across different ethnic groups and LUAD subtypes to ensure clinical reliability and generalizability.

GABARF should serve as a complementary stratification tool to TNM staging rather than a replacement. Our findings indicate that high‐risk patients respond better to first‐line chemotherapy, while low‐risk patients may benefit more from ICIs. However, successful clinical implementation requires establishing standardized operating procedures (SOPs) for sample preparation, data analysis, and result reporting. Prospective, multicenter clinical trials are essential to validate whether GABARF‐guided individualized treatment improves patient outcomes. We propose initial application of GABARF in major oncology centers for clinical trial validation, with gradual accumulation of evidence to support future widespread clinical adoption.

Translating the GABARF model into a real‐time clinical decision‐support tool remains a critical future direction of our work, albeit accompanied by several computational challenges. First, standardizing the bioinformatics pipeline to rapidly process raw clinical RNA‐sequencing data—particularly regarding cross‐platform normalization and real‐time batch‐effect correction against our established training cohort—remains a major technical hurdle. Second, integrating this high‐dimensional transcriptomic signature with existing electronic health record (EHR) systems requires the development of secure, automated, and user‐friendly software interfaces. Such platforms must be capable of generating dynamic risk scores without demanding extensive bioinformatics expertise from front‐line clinicians. Addressing these computational challenges through the development of streamlined, cloud‐based or localized automated pipelines will be the key to bridging the gap between our current bioinformatic multiomics frameworks and bedside precision medicine.

## 5. Limitations

Although this study has conducted comprehensive multi‐omics analyses and developed a GABARF prognostic model, several important limitations warrant acknowledgment. First, the GABARF model was validated using two relatively small external cohorts from similar RNA‐sequencing platforms. Validation using larger, independent datasets from diverse populations and sequencing technologies is necessary to establish generalizability. Second, GABA scores are derived from RNA‐seq data measuring gene expression, which may not accurately reflect functional protein abundance or receptor activity. GABA as a small‐molecule neurotransmitter cannot be directly measured from transcriptomic data. Future studies incorporating direct GABA quantification (LC‐MS) and protein validation (immunohistochemistry) are needed to confirm that RNA‐based GABARF scoring reflects actual GABA pathway activity. Third, although our computational algorithms effectively predicted significant differences in immune infiltration and TME activity between the GABARF risk subgroups, we did not perform direct experimental validations. Future studies incorporating in vitro or in vivo functional immunological assays, such as cytokine release profiling or T‐cell cytotoxicity assays, are warranted to mechanistically elucidate and validate the bidirectional interactions between GABARF and the immune microenvironment in LUAD.

## 6. Conclusion

This study has developed a system pertaining to GABA markers (GABARF) that can function as a potent instrument for predicting prognosis, enabling early prevention, and facilitating personalized diagnosis and treatment in LUAD patients. Moreover, based on GABARgenes, we offer fresh perspectives on the molecular mechanisms underpinning the initiation and progression of LUAD at genomic and transcriptomic levels.

## Author Contributions

Jiangtao You, Tianren Wang, and Qingshi Wang conceptualized and designed the study. Yong Zhang and Rui Zhao performed the data analysis and interpretation. Wei Cui and Huan Chen contributed to the writing and critical revision of the manuscript.

## Funding

No funding was received for this manuscript.

## Disclosure

All authors approved the final version of the manuscript for submission. A preprint of this manuscript has previously been published (Jiangtao You, Tianren Wang, Qingshi Wang, Yong Zhang, Rui Zhao, Wei Cui, and Huan Chen [2024]). Reference entry for the preprint. Jiangtao You, Tianren Wang, Qingshi Wang, Yong Zhang, Rui Zhao, Wei Cui, Huan Chen. Investigation into the Association between Neurotransmitters, Immune Features, and Lung Adenocarcinoma: A Multi‐Omics Approach to the Identification of GABA‐Related Features Employing 101 Combinatorial Machine Learning Computational Frameworks. Preprint. 2024. DOI: 10.21203/rs.3.rs‐4483010/v1.

## Ethics Statement

The authors have nothing to report.

## Consent

The authors have nothing to report.

## Conflicts of Interest

The authors declare no conflicts of interest.

## Supporting Information

Additional supporting information can be found online in the Supporting Information section.

## Supporting information


**Supporting Information** Figure S1. Relative mRNA expression levels of four candidate genes in tumor cells and the control group. Quantitative real‐time PCR (qRT‐PCR) was performed to evaluate the mRNA expression levels of LDHA (A), VDAC1 (B), YWHAZ (C), and EIF4A3 (D) in A549 lung adenocarcinoma cells compared to the control group.

## Data Availability

The datasets generated and analyzed during the present study are available from the corresponding author upon reasonable request.
