# Supplementary figures and images for: Investigation Into the Association Between Neurotransmitters, Immune Features, and Lung Adenocarcinoma: Identifying GABA‐Related Features Using Machine Learning Methods
Source: Stem Cells Int. 2026 May 21;2026:3060138. doi: 10.1155/sci/3060138 (PMC13191820; doi:10.1155/sci/3060138)

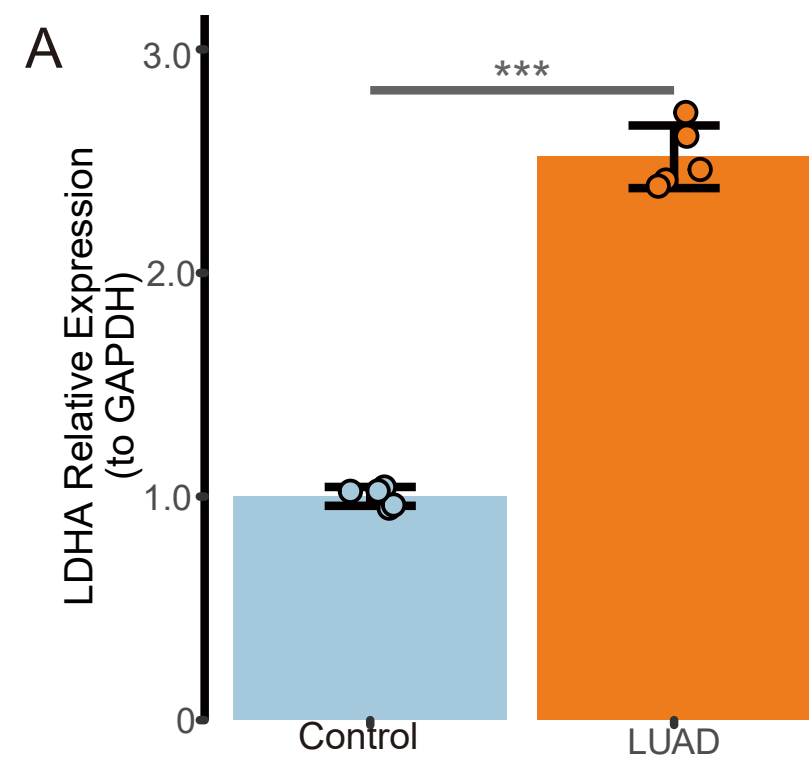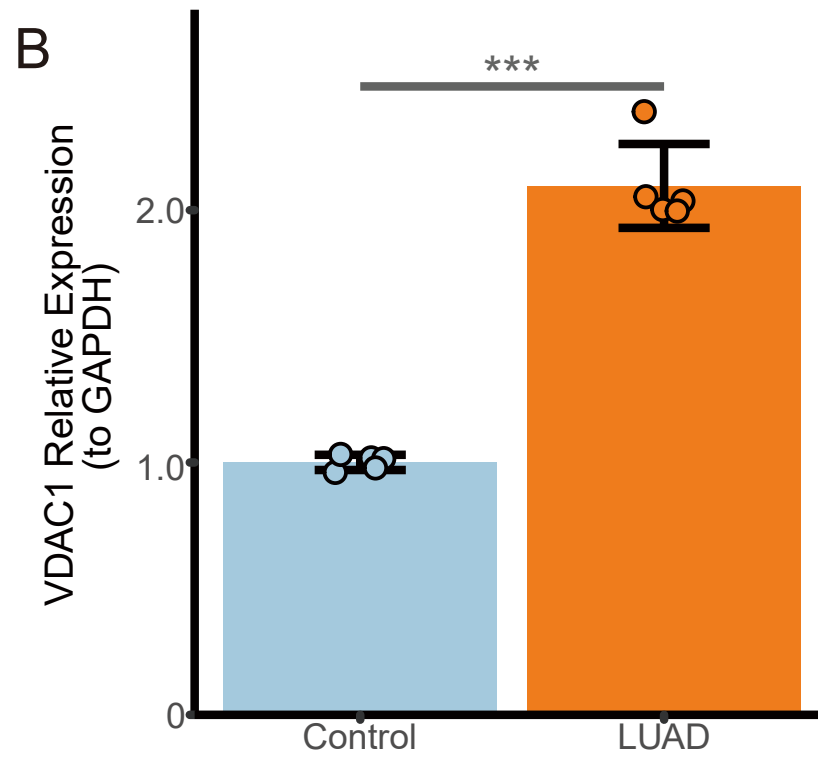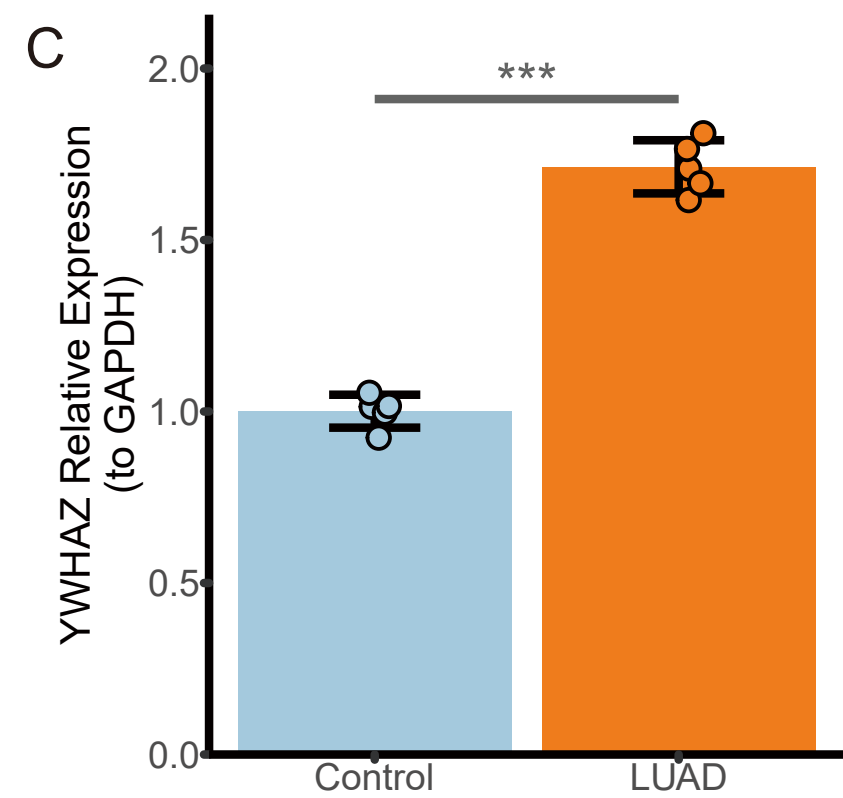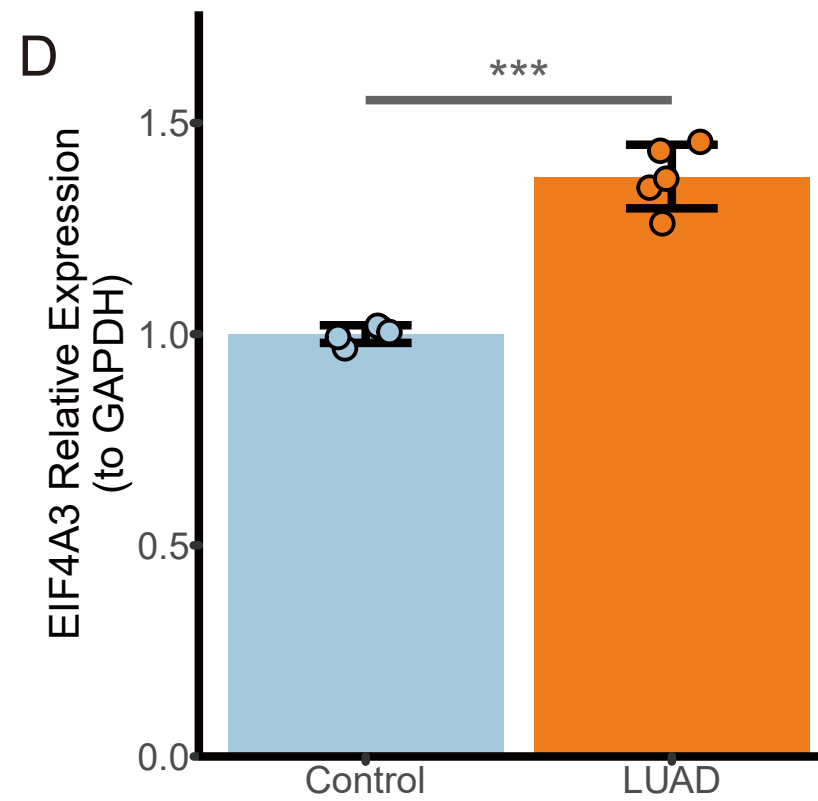

Supplement: Supplementary file 1 — Supporting Information Figure S1. Relative mRNA expression levels of four candidate genes in tumor cells and the control group. Quantitative real‐time PCR (qRT‐PCR) was performed to evaluate the mRNA expression levels of LDHA (A), VDAC1 (B), YWHAZ (C), and EIF4A3 (D) in A549 lung adenocarcinoma cells compared to the control group. [file SCI-2026-3060138-s001.pdf]
